# Anti-Inflammatory Halogenated Monoterpenes from the Red Alga *Portieria hornemannii*

**DOI:** 10.3390/md21090493

**Published:** 2023-09-14

**Authors:** Yuan-Jhong Wu, Tzu-Yin Huang, Chiung-Yao Huang, Chi-Chen Lin, Wei-Lung Wang, Hui-Chi Huang, Shang-Yin Vanson Liu, Chih-Hua Chao, Jyh-Horng Sheu

**Affiliations:** 1 Department of Marine Biotechnology and Resources, National Sun Yat-sen University, Kaohsiung 804, Taiwan; yuan19942@gmail.com (Y.-J.W.); betty8575@yahoo.com.tw (C.-Y.H.); syvliu@mail.nsysu.edu.tw (S.-Y.V.L.); 2 Institute of Biological Chemistry, Academia Sinica, Taipei 115, Taiwan; slime112229@gmail.com; 3 Institute of Biomedical Science, National Chung Hsing University, Taichung 402, Taiwan; lincc@dragon.nchu.edu.tw; 4 Department of Biology, National Changhua University of Education, Changhua 500, Taiwan; wlwang@cc.ncue.edu.tw; 5 Department of Chinese Pharmaceutical Sciences and Chinese Medicine Resources, China Medical University, Taichung 404, Taiwan; hchuang@mail.cmu.edu.tw; 6 School of Pharmacy, China Medical University, Taichung 404, Taiwan; 7 Chinese Medicine Research and Development Center, China Medical University Hospital, Taichung 404, Taiwan; 8 Department of Medical Research, China Medical University Hospital, China Medical University, Taichung 404, Taiwan; 9 Graduate Institute of Natural Products, Kaohsiung Medical University, Kaohsiung 807, Taiwan

**Keywords:** *Portieria hornemannii*, alga, halogenated monoterpenes, anti-inflammatory, STS-method

## Abstract

The chemical investigation of a red alga *Portieria hornemannii* enabled the identification of three new halogenated monoterpenes (**1**–**3**) along with two previously identified metabolites (**4** and **5**). Their structures were determined by spectroscopic analysis and also by utilizing single-crystal diffraction analysis and quantum chemical calculation, as well as by comparison with literature data. Further corrections for dichloro and dibromo carbons using the sorted training set (STS) method were established in this study to significantly improve the accuracy in GIAO ^13^C NMR calculation of compounds **1**–**3**. To discover the potential bioactive metabolites from *P. hornemannii*, the anti-inflammatory activities of all compounds were examined. Compounds **1** and **3**–**5** showed significant anti-inflammatory activity to inhibit the production of pro-inflammatory cytokines in the LPS-stimulated mature dendritic cells.

## 1. Introduction

Halogenated natural products were once dismissed as either artifacts of the isolation process or aberrations in nature [1]. However, it is now recognized that organohalides are synthesized by various organisms, serving diverse roles and often in significant quantities. Red algae, which are widely distributed across the world’s oceans, serve as abundant sources of proteins, fibers, vitamins, physiologically essential fatty acids, and macro- and trace elements. These valuable components can be utilized in food additives and healthcare products [2]. The investigation of red algae constituents has been a subject of study for over four decades [3]. Notably, marine algae from the genera *Plocamium*, *Portieria*, *Laurencia*, and *Ochtodes* have been identified as significant sources of halogenated monoterpenes, encompassing both acyclic and cyclic structural variations [4,5]. Research by Naylor et al. suggests that ocimene acts as the common precursor for all halogenated monoterpenes found in *Plocamium*, while myrcene serves as the shared precursor in *Portieria* and *Ochtodes*, both of which belong to the family Rhizophyllidaceae [6,7]. These compounds are recognized as defensive substances, protecting the algae against herbivores [6]. Moreover, the metabolites derived from marine algae have demonstrated noteworthy potential in the fields of cosmeceuticals and medicine. They exhibit a range of beneficial properties, including skin whitening, anti-aging, anti-inflammatory, antioxidant, antimicrobial, and antitumor activities [7,8].

Halomon, a compound with the chemical name [6*R*-bromo-3*S*-bromomethyl)-7-methyl-2,3,7-trichloro-1-octene], was initially isolated from the red alga *Portieria hornemannii* (Lynbye) collected in the Philippines back in 1992 [9]. Remarkably, halomon exhibited significant differential cytotoxicity against cell lines derived from the brain, kidney, and colon, as determined by the National Cancer Institute’s in vitro human tumor cell line screen [9,10]. Based on its unique cytotoxicity profile, halomon was selected by the NCI for further investigation as a potential candidate for preclinical drug development [9,11]. However, progress in utilizing halomon as an anticancer lead has been hindered by the lack of a reliable natural source and the absence of demonstrated in vivo effects [12]. Efforts to re-isolate halomon from *P. hornemannii* collected from various locations in the Pacific Ocean, including Batan Island in the Philippines, have proven unsuccessful due to variations in terpene content across different sites and over time [13]. Nonetheless, researchers have successfully achieved chemical syntheses of halomon and its analogues, albeit with challenges in achieving precise regio- and stereocontrol [14,15]. Notably, Schlama et al. reported a highly efficient total synthesis of halomon, yielding it and a range of analogues with an overall yield of 13% [16].

**Figure 1:**
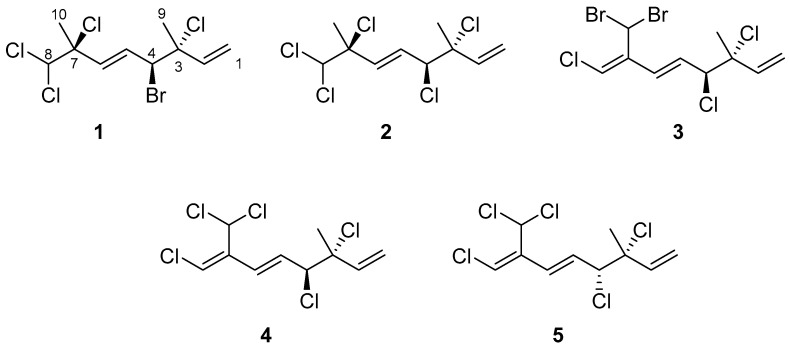
Compounds **1**–**5** isolated from *P. hornemannii*.

A comprehensive literature review revealed that a total of 26 halogenated monoterpenes have been isolated from the red alga *Portieria hornemannii* in previous studies [4,9,17,18,19,20,21]. However, over the past decade, no reports have emerged regarding the bioactive constituents of species from the genus *Portieria*. This knowledge gap, coupled with the inspiration derived from the potential of halomon, motivated us to embark on the exploration and discovery of new bioactive halogenated monoterpenes from the red alga, *P. hornemannii*. In this study, we present the isolation and characterization of three new halogenated monoterpenes (**1**–**3**) (Figure 1), along with two known compounds (**4** and **5**) [22,23], obtained from specimens collected off the coast of Penghu Islands. Additionally, we conducted an anti-inflammatory assay for all isolated compounds in order to identify potential leads for drug development.

## 2. Results

(3*R*,4*S*,5*E*,7*S*)-4-Bromo-3,7,8,8-tetrachloro-3,7-dimethyl-octa-1,5-diene (**1**) was obtained in the form of needle-shaped crystals. Analyzing its HREIMS spectrum, a molecular formula of C_10_H_13_BrCl_4_ was deduced, with four isotope peaks observed at *m*/*z* 351, 353, 355, and 357, exhibiting approximate intensities of 4:10:8:3, respectively [24] (see Figures S1 and S2 in Supplementary Materials). The ^13^C NMR and DEPT spectra of compound **1** displayed 10 carbon signals, encompassing two methyl groups, one methylene group, five methine groups, and two quaternary carbons, as indicated in Table 1. Additionally, distinctive signals corresponding to a mono-substituted double bond (*δ*_H_ 5.27, d, *J* = 10.8 Hz; 5.39, d, *J* = 17.2 Hz; 6.05, dd, *J* = 17.2, 10.8 Hz; *δ*_C_ 116.4, CH_2_; 139.8, CH), a *trans* double bond (*δ*_H_ 6.16, dd, *J* = 15.2, 9.6 Hz; 6.04, d, *J* = 15.2 Hz; *δ*_C_ 130.4, CH; 133.8, CH), two chlorine-bearing quaternary carbons (*δ*_C_ 71.3, C; 71.6, C), a bromine-bearing methine (*δ*_H_ 4.65, *d*, *J* = 9.6 Hz; *δ*_C_ 60.4, CH) [7], a dichloromethyl group (*δ*_H_ 5.79, s; *δ*_C_ 78.4, CH), and two methyl groups attached to halogenated quaternary carbons (*δ*_H_ 1.81, s; 1.89, s; *δ*_C_ 25.6, CH_3_; 24.9, CH_3_) were observed. Based on these findings, the structure of compound **1** was suggested to be a halogenated acyclic monoterpene.

**Figure 2:**
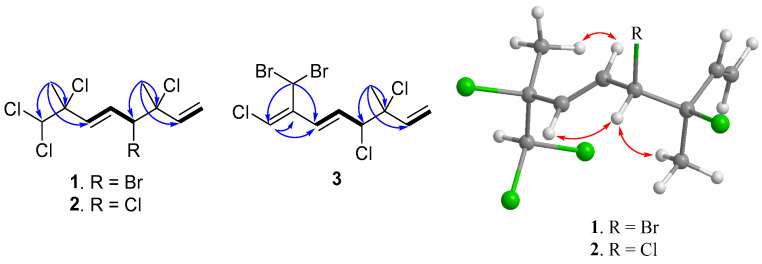
COSY (Bold) and selective HMBC (arrow) of **1**–**3** and selective NOE correlations of **1** and **2** (double-headed arrow).

The determination of the planar structure of compound **1** was achieved through a comprehensive analysis of COSY and HMBC spectra. The COSY correlations unveiled two separate spin systems, namely H_2_-1/H-2 and H-4/H-5/H-6. Moreover, the HMBC correlations observed from H_3_-9 to C-2, C-3, and C-4, along with correlations from H_3_-10 to C-6, C-7, and C-8, effectively connected the two spin systems and subsequently confirmed the planar structure of compound **1**, as depicted in Figure 2 (see Figures S3–S9 in Supplementary Materials).

**Figure 3:**
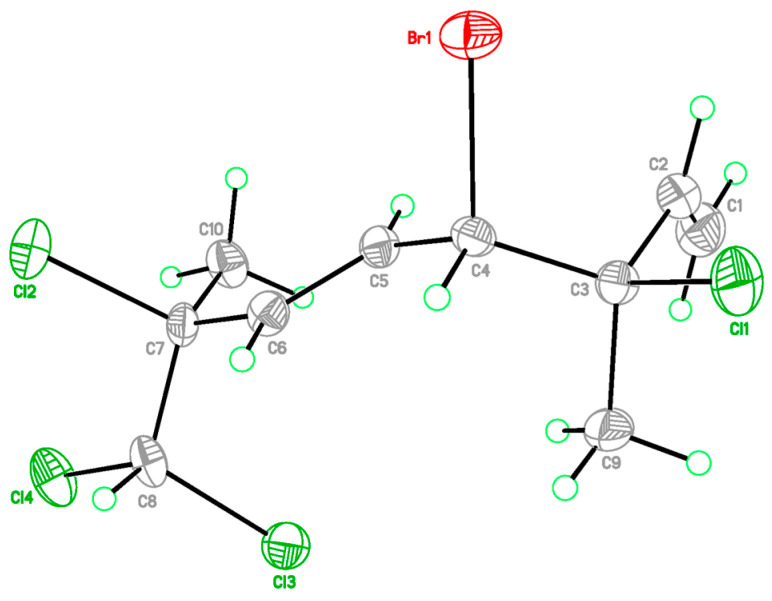
X-ray ORTEP drawing of **1**.

**Figure 4:**
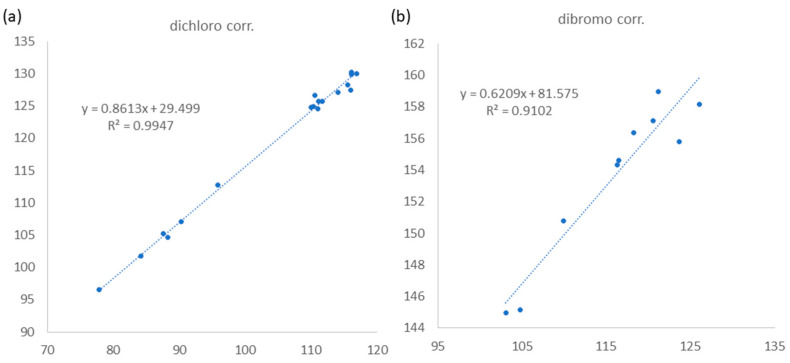
Linear relationship between *I*_retro_ (y axis, experimental chemical shifts retroconverted into pseudo experimental shielding tensors) and *I*_calcd_ (x axis, shielding tensors from computational calculation) for corrections of (**a**) dichloro and (**b**) dibromo carbons following STS method.

The relative configurations at C-3 and C-4 were determined by a comparison of ^13^C NMR data of the C-3 methyl shift with those of related analogues in literature [7], in which the methyl shift of *δ*_C_ 25–26 was suggested for 3,4-*erythro* compound and *δ*_C_ 28 for 3,4-*threo* isomer. Consequently, a 3,4-*erythro* configuration was assigned for compound **1** (*δ*_C_ 25.6, C-9). The relative configuration at C-7 was determined by the application of the sorted training set (STS) method allowed for theoretical NMR calculations. This protocol was utilized to enhance the precision and dependability of structure assignment, particularly in cases involving carbons bearing heavy atoms (Cl, S, Br, I, etc.) [25]. The calculation data (uncorrected data, Table 2) could not show a conclusive result, with *P*_rel_ probabilities of 43.55% and 56.45% for 3R*,4S*,7S*-**1** and 3R*,4S*,7R*-**1**, respectively. This was ascribed to the lack of corrections for dichloro carbons in the STS method. Thus, 18 dichloro model compounds from the literature were selected and their NMR data were computed following the STS protocol to obtain a correction formula (*I_corr_* = 0.8613*I_cald_* + 29.499, *I_calcd_*: calculated shielding tensor). Using the correction formula for dichloro carbon (C-8) in **1**, the mean absolute error (MAE) and root mean square (rms) values of 3R*,4S*,7S*-**1** are as low as 1.07 and 1.33, respectively, which are much better than the uncorrected data. Furthermore, 3R*,4S*,7S*-**1** was suggested to be the correct structure with the *P*_rel_ probability of 77.33%, which is much higher than that of 3R*,4S*,7R*-**1** (22.67%). To further confirm the structure and determine the absolute configuration of **1**, a single-crystal X-ray diffraction analysis was performed. The X-ray crystallography data from **1** with a Flack parameter of 0.001(9) demonstrated its structure and absolute configuration to be 3*R*,4*S*,7*S* (Figure 3).

(3R*,4S*,5*E*)-3,4,7,8,8-Pentachloro-3,7-dimethyl-octa-1,5-diene (**2**) was isolated as a colorless oil. The high-resolution chemical ionization mass spectrometry (HRCIMS) analysis of its spectrum revealed the molecular formula to be C_10_H_13_Cl_5_ (see Figures S10 and S11 in Supplementary Materials). The ^13^C NMR and DEPT spectra of compound **2** revealed the presence of 10 carbon signals, encompassing two methyl groups, one methylene group, five methine groups, and two quaternary carbons, as outlined in Table 1. The findings, combined with a thorough comparison of NMR data between **1** and **2**, indicated that **2** is a polychlorinated monoterpene closely related to **1** (as shown in Table 1). The most significant disparity was observed for the brominated methine in **1** (δ_C_ 60.4, C-4), where it was substituted by a chlorine atom in **2** (δ_C_ 67.9, C-4) [7]. The HMBC correlation between H_3_-9 (δ_H_ 1.75, s) and C-2 (δ_C_ 139.5), C-3 (δ_C_ 71.7), as well as C-4 (δ_C_ 67.9), further supported the aforementioned deduction (refer to Figure 2 and Figures S12–S18 in Supplementary Materials). By examining the carbon resonance of the C-3 methyl group of **2** (δ_C_ 24.9, C-9), the configuration of 3,4-*erythro* was established [7]. Similar to compound **1**, the relative configuration at C-7 of **2** was determined using the STS method with the established dichloro correction. The 3R*,4S*,7S*-configuration was suggested for **2** due to its *P*_rel_ probability of 71.24% (Table 3). Moreover, the NOE correlations of **2** showed close similarity to those of **1**, conforming the same configurations of both compounds (Figure 2).

(3*S*,4*R*,5*E*,7*Z*)-3,4,8-trichloro-7-dibromomethyl-3-methyl-octa-1,5,7-triene (**3**) was found to be a colorless oil with a molecular formula of C_10_H_11_Br_2_Cl_3_, as suggested by analysis of HREIMS and NMR spectra (see Figures S19–S29 in Supplementary Materials), revealing the presence of the three chlorines and two bromine atoms. Upon analysis of the ^13^C NMR and DEPT spectra, it was determined that compound **3** exhibited 10 carbon signals. These signals encompassed one methyl group, one sp^2^ methylene group, four sp^2^ methine groups, two sp^3^ methine groups, one sp^3^ quaternary carbon, and one sp^2^ quaternary carbon, as outlined in Table 1. A comparison of the NMR data of **3**, measured in acetone-*d*_6_, with those of **4** (Table S1, see Supplementary data) indicated that they have the same fragment from C-1 to C-5, which was confirmed by interpretations of spin systems of H-1/H-2 and H-4/H-5/H-6 from COSY spectra was well as by the HMBC correlations from H_3_-9 to C-2, C-3, and C-4 (Figure 2). The comparison also allowed the confirmation of a 3,4-*erythro* configuration for the dichloro substituents [*δ*_C_ 73.4 (C-3), 70.0 (C-4), and 26.1 (C-9) for **3**; *δ*_C_ 73.4 (C-3), 70.0 (C-4), and 26.0 (C-9) in acetone-*d*_6_ for **5**] (Table S1). A dibromomethyl group attached at C-9 was assigned by the NMR chemical shift [*δ*_C_ 33.7 (C-10), *δ*_H_ 6.92 (H-10) in CDCl_3_] (Table 1). The carbon attached to a bromine or chlorine atom could deshield the chemical shift and the effect for the chlorine atom is generally larger. Similarly, the presence of a remaining sp^2^ methine at *δ*_C_ 117.2 (C-8) suggested that chlorine is attached at this position. This conclusion is drawn by comparing the data with the existing literature, which indicates that vinyl bromide is typically observed at a chemical shift of approximately *δ*_C_ 108–110 ppm [7], while vinyl chloride is typically observed at 118–124 ppm [7,26]. A 15.2 Hz of ^1^H–^1^H coupling constant gave evidence for *E*-geometry of the 5,6-double bond in **3** (see Table S1 in Supplementary Materials). Based on the result reported by Mynderse and Faulkner as well as the synthetic analogues [22,26], the geometry of the 7,8-double bond was found to be associated with the chemical shift of H-6 and it can be concluded that, in the 5,7-diene system with C-8 halogen *trans* to H-6, the proton chemical shift of H-6 is found at approximately *δ*_H_ 6.2–6.3 (in CDCl_3_). However, in the *cis* analogue, the shift is found at approximately *δ*_H_ 6.5–6.8 (in CDCl_3_). The H-6 proton NMR shift of compound **3**, found at *δ*_H_ 6.39 in CDCl_3_ (Table 1), indicated that its 7,8-double bond has *Z* geometry. Moreover, the proton shift of the H-6 in compound **3**, measured in acetone-*d*_6_, was found to be at *δ*_H_ 6.55. This value is in close proximity to the known compounds **4** (*δ*_H_ 6.52) and **5** (*δ*_H_ 6.53) as shown in Table S1. These findings suggest the presence of a 7*Z* double bond in compound **3.** To provide further confirmation, the application of the STS method for theoretical NMR calculation was performed on **3**. The result (Table 4) showed high deviation at dibromo methine carbon C-10 (∆ = 19.17 ppm for 7*Z* and 23.29 ppm for 7*E*, ∆ = *δ_calcd_* − *δ_exptl_*), which can be explained by the lack of corrections for dibromo carbons in the STS strategy. Thus, we selected 10 dibromo model compounds from literature and computed their NMR data following the STS protocol to obtain a correction formula (*I_corr_* = 0.6209*I_calcd_* + 81.575, *I_calcd_*: calculated shielding tensor) (Figure 4). Using the correction formula for dibromo carbon resulted in a significant improvement in accuracy for C-10 (Table 4). Furthermore, replacement of C-10 data by the corrected shielding tensor would show good results (*P*_rel_ probability, 100%), which confirmed the 7*Z* configuration for **3**.

To date, more than 100 halogenated monoterpenes have been discovered from different organisms [27]. Some halogenated monoterpenes have been demonstrated to show cytotoxic [9,17,18], antimalarial [28], and insecticidal activities [29,30,31]. In the present study, we isolated four new halogenated monoterpenoids, and two reported isolates. To discover any potential drug leads, all of the metabolites were examined with the anti-inflammatory assay. Compounds **1**, **4**, and **5** were found to significantly reduce the production of TNF-α in the LPS-stimulated dendritic cells with IC_50_ values of 2.5 ± 0.4, 6.2 ± 1.1, and 10.6 ± 1.3, respectively (Table 5), which is more potent than the positive control, quercetin (IC_50_ = 23.1 ± 5.2).

**Figure 5:**
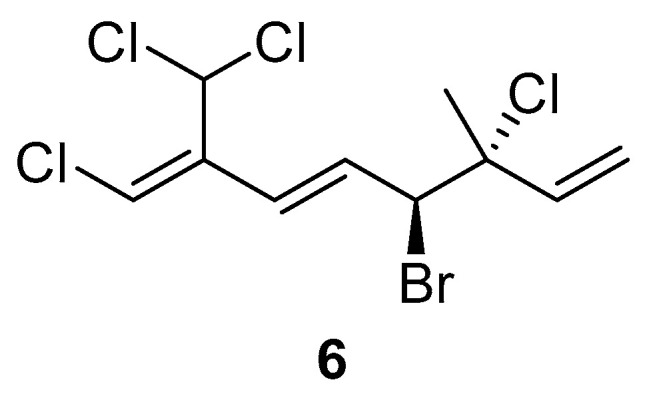
The proposed structure **6** with potential bioactivity.

The comparison of anti-inflammatory activity between **1** and **2** indicated that the bromine atom at C-4 seems to be essential for the biological activity. Additionally, in the case of **3**–**5**, compound **3** with two bromine atoms at C-10 did not show any activity in the dose test. However, two chlorine atoms attached at C-10 seem to be responsible for the biological activity. Based on the above evidence, an undiscovered halogenated monoterpene **6** (Figure 5) that might show potential anti-inflammatory activity is waiting for discovery in the future.

## 3. Materials and Methods

### 3.1. General Experimental Procedures

Measurements of optical rotations were conducted for compounds **1**–**5** utilizing a JASCO P-1020 polarimeter (JASCO Corporation, Tokyo, Japan), and their NMR spectra were recorded on Varian 400 MR FT-NMR and Varian Unity INOVA500 FT-NMR (Varian Inc., Palo Alto, CA, USA) instruments at 400 MHz and 500 MHz for ^1^H and 100 MHz and 125 MHz for ^13^C in CDCl_3_ and acetone-*d*_6_ at 25 °C. Electron impact mass spectrometry (EIMS) and HREIMS spectra were afforded on the magnetic sector mass spectrometry (JEOL, JMS-700, Tokyo, Japan). A normal-phase column packed with silica gel (Merck, 230–400 mesh) was utilized in the experiment, while thin-layer chromatography (TLC) with pre-coated silica gel plates (Merck, Kieselgel 60 F-254, 0.2 mm, Merck, Darmstadt, Germany) was employed for analyzing each isolated fraction. Additionally, high-performance liquid chromatography was conducted using a Supelco C18 column (250 × 21.2 mm, 5 μm; Supelco, Bellefonte, PA, USA) and a Hitachi L-2455 HPLC apparatus.

### 3.2. Plant Material

The red alga *P. hornemannii* (wet weight: 1.53 kg) was collected by hand using SCUBA from the sea area near the Pescadores Islands in 2012. Until the commencement of the experiment, the material sample had been kept at the Department of Marine Biotechnology and Resources, National Sun Yat-sen University. The red alga was identified by Dr. Wei-Lung Wang.

### 3.3. Extraction and Isolation

A solvent-free sample of *P. hornemannii* weighing 720.4 g was subjected to exhaustive extraction with ethyl acetate (1L × 5). The resulting organic extract was concentrated, yielding an oily residue weighing 4.45 g. Subsequently, the oily residue was chromatographed using silica gel column chromatography, employing stepwise elution with ethyl acetate in *n*-hexane (0–100%) and then with acetone in methanol (0–100%), resulting in the separation of 27 fractions. Fraction 1, obtained by elution with *n*-hexane (100%), was further purified using RP-18 reversed-phase silica gel column chromatography with acetonitrile/H_2_O (3:1), resulting in the isolation of subfractions (A–E). Subfraction 1-C was further subjected to purification via RP-HPLC using MeOH/H_2_O (4.5:1) at a flow rate of 5 mL/min, leading to the isolation of compound **3** (2.4 mg). Similarly, subfraction 1-D was purified by RP-HPLC using MeOH/H_2_O (4.5:1) at a flow rate of 5 mL/min, yielding compound **4** (8.9 mg). On the other hand, fraction 2 was eluted with *n*-hexane/ethyl acetate (8:1) and then subjected to chromatography over an RP-18 reversed-phase silica gel column using acetonitrile/H_2_O (4.5:1), resulting in the separation of six subfractions (A–F). Subfraction 2-D was isolated through HPLC with MeOH/H_2_O (5:1), leading to the isolation of compound **1** (16.3 mg), while subfraction 2-E was purified in a similar manner using MeOH/H_2_O (4.5:1), resulting in the isolation of compounds **2** (5.3 mg) and **5** (15.8 mg).

(3*R*,4*S*,5*E*,7*S*)-4-Bromo-3,7,8,8-tetrachloro-3,7-dimethyl-octa-1,5-diene (**1**): needle-shaped crystal, ${\left[\alpha \right]}_{D}^{26}$ − 9.7 (*c* 0.38, CHCl_3_); for ^13^C and ^1^H data see Table 1; EIMS *m*/*z* 351 ${\left[M\right]}^{+}$; HREIMS *m*/*z* 351.8955 ${\left[M\right]}^{+}$ (calculated for C_10_H_13_^79^Br^35^Cl_4_: 351.8950).

(3R*,4S*,5*E*)-3,4,7,8,8-Pentachloro-3,7-dimethyl-octa-1,5-diene (**2**): colorless oil, ${\left[\alpha \right]}_{D}^{26}$ +16.4 (*c* 0.73, CHCl_3_), for ^13^C and ^1^H data see Table 1; HRCIMS *m*/*z* 274.9739 ${[M–\mathrm{Cl}]}^{+}$ calculated for C_10_H_13_^35^Cl_3_^37^Cl: 274.9737).

(3*S*,4*R*,5*E*,7*Z*)-3,4,8-Trichloro-7-dibromomethyl-3-methyl-octa-1,5,7-triene (**3**): colorless oil, ${\left[\alpha \right]}_{D}^{26}$ + 45.0 (*c* 0.2, CHCl_3_), for ^13^C and ^1^H data see Table 1; EIMS *m*/*z* 360 ${[M–\mathrm{Cl}]}^{+}$; HREIMS *m*/*z* 360.8567 ${[M–\mathrm{Cl}]}^{+}$ calculated for C_10_H_11_^79^Br_2_^35^Cl^37^Cl: 360.8579).

### 3.4. Crystallographic Data for ***1***

C_10_H_13_BrCl_4_, M = 354.91, Orthorhombic, space group *P*2_1_2_1_2_1_, *a* = 7.6488(4) Å, *b* = 10.4285(6) Å, *c* = 17.5409(11) Å, α = 90°, β = 90°, γ = 90°, *V* = 1399.16(14) Å^3^, *T* = 150(2) K, *Z* = 4, μ(Cu Kα) = 3.669 mm^–1^, *D*_calcd_ = 1.685 Mg/m^3^, crystal size 0.20 × 0.15 × 0.10 mm^3^, *F*(000) = 704, 5356 reflections measured, 3058 independent reflections (*R*_int_ = 0.0359). The final *R*_1_ was 0.0396 (*I* > 2σ(*I*)), and *wR*_2_ was 0.0630. The final *R*_1_ was 0.0521 (all data) and *wR*_2_ was 0.0688 (all data). The goodness of fit on *F*^2^ was 0.975. Flack parameter = 0.001(9). Crystallographic data for **1** have been deposited at the Cambridge Crystallographic Data Center (deposition CCDC number 2246798). Copies of the data can be obtained free of charge via www.ccdc.cam.ac.uk (accessed on 7 March 2023).

### 3.5. Quantum Chemical Calculations

Two possible candidates for each compound were subjected to conformational searches on GMMX add-on of GaussView 6 (Semichem Inc., Shawnee Mission, KS, USA). This was followed by preliminary optimizations of conformers within a 5 kcal/mol energy window at the B3LYP/6-31G(d) level of theory using Gaussian 16 software (Gaussian, Inc., Wallingford, CT, USA) [32]. After removing duplicates, further optimizations and frequency calculations were computed at the B3LYP-D3(BJ)/TZVP level, employing the IEFPCM solvent mode with CHCl_3_ as the solvent. Structures with imaginary frequencies were excluded from subsequent calculations. Theorical shielding tensors were calculated at the IEFPCM (CHCl_3_)/ωB97xD/6-31G(d) level of theory. The data were weighted according to Boltzmann population using Gibbs free energy and subjected to STS analysis [25]. All calculations were performed with the “g09defdaults” keyword in Gaussian 16 to ensure consistent results, as suggested in the literature [25]. A total of 18 and 10 model compounds were selected from the literatures for dichloro- and dibromo-carbon shift corrections, respectively (Supplementary Materials: Figures S30 and S31). The calculated shielding tensors for the model compounds were obtained following the same protocol as described above. The experimental chemical shifts were retroconverted into pseudo shielding tensors (*I*_retro_) by the equation: I*_retro_* = (199.81 − *δ_exptl_*)/1.0266 [25] and then regressed with calculated shielding tensors (I*_calcd_*) to obtain the correction linear equation as shown in Figure 4.

### 3.6. Anti-Inflammatory Assay

In this study, the viability of D.C. cells was evaluated at a concentration of 100 μg/mL using a cell-counting-kit assay (CCK-8) obtained from Dojindo Molecular Technologies, Inc. (Kumamoto, Japan), which measured absorbance at a wavelength of 450 nm, following the methodology outlined by Chung et al. [33]. The expression of the pro-inflammatory cytokine, TNF-α, in the culture supernatant was screened using the dendritic cells ELISA kit, as described in a previous study [34]. The cytotoxicity of quercetin against DCs was measured in this study as a positive control, with IC_50’s_ of 78.8 ± 7.3 μM. Additionally, quercetin exhibited inhibitory effects on the reduction of TNF-α expression in LPS-induced DCs, with IC_50’s_ of 23.1 ± 5.2.

## 4. Conclusions

The present investigation on the red alga *P. hornemannii* led to the characterization of three new halogenated monoterpenes and two reported analogues, of which the structures were elucidated based on the analysis of their MS and NMR spectroscopic data. We also determined the absolute configuration of **1** through its X-ray crystallography data. Compound **3**, which contains a rare 1-chloro-3,3-dibromoprop-1-en-2-yl moiety, has been found for the first time in *P. hornemannii*. In the anti-inflammatory assay, compounds **1**, **4**, and **5** were demonstrated to significantly reduce the TNF-α in the LPS-stimulated dendritic cells. Evaluation of the result of biological assays concluded that the structural motifs with C-4 bromo and C-10 dichloro substituents in acyclic monoterpenes are important for anti-inflammatory activity. Our research has once again confirmed that the red alga *P. hornemannii* is a good source of bioactive halogenated monoterpenes.

## Figures and Tables

**Table 1:** ^1^H (400 MHz) and ^13^C NMR (100 MHz) spectroscopic data for **1**–**3** in CDCl_3_.

	1		2		3	
Position	*δ*_C_ (type)	*δ*_H_ (*J* in Hz)	*δ*_C_ (type)	*δ*_H_ (*J* in Hz)	*δ*_C_ (type)	*δ*_H_ (*J* in Hz)
1	116.4 (CH_2_)	5.27 d (10.8)	116.5 (CH_2_)	5.28 d (10.8)	116.5 (CH_2_)	5.31 d (10.8)
		5.39 d (17.2)		5.40 d (17.2)		5.43 d (16.8)
2	139.8 (CH)	6.05 dd (17.2, 10.4)	139.5 (CH)	6.06 dd (17.2, 10.8)	139.5 (CH)	6.11 dd (16.8, 10.8)
3	71.3 (C)		71.7 (C)		71.9 (C)	
4	60.4 (CH)	4.65 d (9.6)	67.9 (CH)	4.51 dd (5.6, 2.0)	68.8 (CH)	4.58 m
5	130.4 (CH)	6.16 dd (15.2, 9.6)	129.8 (CH)	6.10 overlapped	130.2 (CH)	6.39 overlapped
6	133.8 (CH)	6.04 d (15.2)	134.5 (CH)	6.10 overlapped	128.3 (CH)	6.39 overlapped
7	71.6 (C)		71.8 (C)		138.5 (C)	
8	78.4 (CH)	5.79 s	78.4 (CH)	5.79 s	117.2 (CH)	6.15 s
9	25.6 (CH_3_)	1.81 s	24.9 (CH_3_)	1.75 s	25.1 (CH_3_)	1.80 s
10	24.9 (CH_2_)	1.89 s	25.0 (CH_3_)	1.89 s	33.7 (CH)	6.92 s

**Table 2:** GIAO ^13^C NMR calculations for 3R*,4S*,7S* and 3R*,4S*,7R* isomers of **1** following the STS method.

		3R*,4S*,7S*-1				3R*,4S*,7R*-1			
Position	Exptl.	Uncorr. ^a^	Dev.	Corr. ^b^	Dev.	Uncorr. ^a^	Dev.	Corr. ^b^	Dev.
1	116.4	116.17	0.23	116.66	0.26	116.20	0.20	116.69	0.29
2	139.8	138.00	1.80	138.44	1.36	137.88	1.92	138.32	1.48
3	71.3	73.47	2.17	74.04	2.74	73.49	2.19	74.07	2.77
4	60.4	58.67	1.73	59.27	1.13	58.65	1.75	59.25	1.15
5	130.4	131.02	0.62	131.48	1.08	131.08	0.68	131.53	1.13
6	133.8	133.01	0.79	133.47	0.33	133.11	0.69	133.56	0.24
7	71.6	72.25	0.65	72.82	1.22	72.37	0.77	72.94	1.34
8	78.4	81.33	2.93	76.4	2.00	81.11	2.71	76.18	2.22
9	25.6	24.29	0.61	25.06	0.54	24.24	1.36	24.92	0.68
10	24.9	24.39	1.21	24.96	0.06	24.47	0.43	25.15	0.25
		MAE	1.27	MAE	1.07	MAE	1.27	MAE	1.16
		rms	1.51	rms	1.33	rms	1.50	rms	1.41
		*P*_mean_	21.71%	*P*_mean_	28.75%	*P*_mean_	22.28%	*P*_mean_	25.43%
		*P*_rel_	43.55%	*P*_rel_	77.33%	*P*_rel_	56.45%	*P*_rel_	22.67%

^a^ The calculated chemical shifts were obtained following the STS protocol. ^b^ The chemical shifts were obtained following the STS protocol except that the chemical shifts of C-8 (dichloromethine) were obtained from a corrected shielding tensor using the formula (*I_corr_* = 0.8613*I_cald_* + 29.499), and subsequently converted to chemical shift using the linear equation (*δ* = −1.0266*I_corr_* + 199.81) from the reported STS protocol.

**Table 3:** GIAO ^13^C NMR calculations for 3R*,4S*,7S* and 3R*,4S*,7R* isomers of **2** following the STS method.

		3R*,4S*,7S*-2				3R*,4S*,7R*-2			
Position	Exptl.	Uncorr. ^a^	Dev.	Corr. ^b^	Dev.	Uncorr. ^a^	Dev.	Corr. ^b^	Dev.
1	116.5	116.29	0.21	116.78	0.28	116.32	0.18	116.80	0.30
2	139.5	138.13	1.37	138.57	0.93	138.01	1.49	138.44	1.06
3	71.7	73.56	1.86	74.13	2.43	73.58	1.88	74.16	2.46
4	67.9	65.17	2.73	65.77	2.13	65.15	2.75	65.74	2.16
5	129.8	131.15	1.35	131.60	1.80	131.21	1.41	131.66	1.86
6	134.5	133.14	1.36	133.59	0.91	133.24	1.26	133.68	0.82
7	71.8	72.33	0.53	72.91	1.11	72.46	0.66	73.04	1.24
8	78.4	81.43	3.03	76.49	1.91	81.20	2.80	76.28	2.12
9	24.9	24.45	0.45	25.13	0.23	24.29	0.61	24.99	0.09
10	25.0	24.35	0.65	25.03	0.03	24.52	0.48	25.22	0.22
		MAE	1.35	MAE	1.18	MAE	1.35	MAE	1.23
		rms	1.63	rms	1.43	rms	1.61	rms	1.49
		*P*_mean_	19.12%	*P*_mean_	25.80%	*P*_mean_	19.84%	*P*_mean_	23.57%
		*P*_rel_	40.80%	*P*_rel_	71.24%	*P*_rel_	59.20%	*P*_rel_	28.76%

^a^ The calculated chemical shifts were obtained following the STS protocol. ^b^ The chemical shifts were obtained following the STS protocol except that the chemical shifts of C-8 (dichloromethine) were obtained from a corrected shielding tensor using the formula (*I_corr_* = 0.8613*I_cald_* + 29.499), and subsequently converted to chemical shift using the linear equation (*δ* = −1.0266*I_corr_* + 199.81) from the reported STS protocol.

**Table 4:** GIAO ^13^C NMR calculations for 7*Z* and 7*E* isomers of **3** following the STS method.

		7*Z*-3				7*E*-3			
Position	Exptl.	Uncorr. ^a^	Dev.	Corr. ^b^	Dev.	Uncorr. ^a^	Dev.	Corr. ^b^	Dev.
1	116.5	114.27	2.23	115.47	1.03	113.66	2.84	115.13	1.37
2	139.5	136.26	3.24	136.28	3.22	136.23	3.27	136.57	2.93
3	71.9	68.55	3.35	72.19	0.29	67.95	3.95	71.67	0.23
4	68.8	63.28	5.52	67.20	1.60	63.11	5.69	67.07	1.73
5	130.2	129.60	0.60	129.98	0.22	132.11	1.91	132.66	2.46
6	128.3	127.95	0.35	128.42	0.12	122.55	5.75	123.57	4.73
7	138.5	138.58	0.08	138.48	0.02	136.24	2.26	136.58	1.92
8	117.2	121.41	4.21	122.23	5.03	125.00	7.80	125.90	8.70
9	25.1	16.93	8.17	23.32	1.78	15.86	9.24	22.15	2.95
10	33.7	52.87	19.17	36.13	2.43	56.99	23.29	38.40	4.70
				MAE	1.57			MAE	3.17
				rms	2.20			rms	3.90
				*P*_mean_	11.24%			*P*_mean_	0.51%
				*P*_rel_	100.00%			*P*_rel_	0.00%

^a^ The calculated chemical shifts were obtained following the STS protocol. ^b^ The chemical shifts were obtained following the STS protocol except that the chemical shifts of C-10 (dibromomethine) were obtained from a corrected shielding tensor using the formula (*I_corr_* = 0.6209*I_cald_* + 81.575), and subsequently converted to chemical shift using the linear equation (*δ* = −1.0266*I_corr_* + 199.81) from the reported STS protocol.

**Table 5:** Anti-inflammatory result of compounds **1**–**5**.

	Anti-Inflammatory Activity (IC_50_) (μM)	
Compound	Cell Viability (%) (DCs)	TNF-α Expression (LPS/DCs)
**1**	47.0 ± 2.1	2.5 ± 0.4
**2**	88.8 ± 8.1	>100
**3**	80.2 ± 8.3	>100
**4**	51.1 ± 2.2	6.2 ± 1.1
**5**	49.1 ± 2.7	10.6 ± 1.3

Data presented as mean ± S.D. and experiments were performed in triplicate.

## Data Availability

Data from the present study are available in the article and Supplementary Materials.

## Supplementary Materials

The following supporting information can be downloaded at: https://www.mdpi.com/article/10.3390/md21090493/s1, Figures S1 and S2: mass spectra of **1**, Figures S3–S9: NMR spectra of **1** in CDCl_3_, Figures S10 and S11: mass spectra of **2**, Figures S12–S18: NMR spectra of **2** in CDCl_3_, Figures S19 and S20: mass spectra of **3**, Figures S21 and S22: NMR spectra of **3** in CDCl_3_, Figures S23–S29: NMR spectra of **3** in acetone-*d*_6_, Figures S30–S32: The STS-method data sheets of compounds **1**–**3**. Table S1: ^1^H and ^13^C NMR spectroscopic data of **3**, **4**, and **5** in acetond-*d_6_*, Table S2: Comparison of specific optical rotations and selected NMR data of synthetic halogenated monoterpenes.
