# Supplementary material for: Anti-Inflammatory Halogenated Monoterpenes from the Red Alga Portieria hornemannii
Source: Mar Drugs. 2023 Sep 14;21(9):493. doi: 10.3390/md21090493 (PMC10533049; doi:10.3390/md21090493)
Supplement: Supplementary file 1 [file marinedrugs-21-00493-s001.zip › marinedrugs-2613313-supplementary.pdf]

Anti-inflammatory Halogenated Monoterpenes from the Red Alga *Portieria hornemannii*

Yuan-Jhong Wu [1,†], Tzu-Yin Huang [2,†], Chiung-Yao Huang [1], Chi-Chen Lin [3], Wei-Lung Wang [4], Hui-Chi Huang [5], Shang-Yin Vanson Liu [1], Chih-Hua Chao [6,7,*] and Jyh-Horng Sheu [1,8,9,*]

[1] Department of Marine Biotechnology and Resources, National Sun Yat-sen University, Kaohsiung 804, Tai-wan; yuan19942@gmail.com (Y.J.W.); betty8575@yahoo.com.tw (C.Y.H.); syvliu@mail.nsysu.edu.tw (S.Y.V.L.)

[2] Institute of Biological Chemistry, Academia Sinica, Taipei 115, Taiwan; slime112229@gmail.com (T.Y.H.)

[3] Institute of Biomedical Science, National Chung Hsing University, Taichung 402, Taiwan; lincc@dragon.nchu.edu.tw (C.C.L.)

[4] Department of Biology, National Changhua University of Education, Changhua 500, Taiwan; wlwang@cc.ncue.edu.tw (W.L.W.)

[5] Department of Chinese Pharmaceutical Sciences and Chinese Medicine Resources, China Medical University, Taichung, 404, Taiwan; hchuang@mail.cmu.edu.tw (H.C.H.)

[6] School of Pharmacy, China Medical University, Taichung 404, Taiwan

[7] Chinese Medicine Research and Development Center, China Medical University Hospital, Taichung 404, Taiwan

[8] Department of Medical Research, China Medical University Hospital, China Medical University, Taichung 404, Taiwan

[9] Graduate Institute of Natural Products, Kaohsiung Medical University, Kaohsiung 807, Taiwan

† These authors contributed equally to this work.

* Correspondence: chchao@mail.cmu.edu.tw (C.H.C.); Tel: +886-4-22053366 (ext. 5157) (C.H.C.); Fax: +886-4-22078083 (C.H.C.); sheu@mail.nsysu.edu.tw (J.H.S.); Tel.: +886-7-525-2000 (ext. 5030) (J.H.S.); Fax: +886-7-525-5020 (J.H.S.)

Figure S1. (+)-EIMS spectrum of **1**.
Figure S2. (+)-HREIMS spectrum of **1**.
Figure S3. ^{1}H NMR spectrum of **1** in $CDCl_3$.
Figure S4. ^{13}C NMR spectrum of **1** in $CDCl_3$.
Figure S5. DEPT135 (upper) and DEPT90 (lower) spectra of **1** in $CDCl_3$.
Figure S6. HSQC spectrum of **1** in $CDCl_3$.
Figure S7. COSY spectrum of **1** in $CDCl_3$.
Figure S8. HMBC spectrum of **1** in $CDCl_3$.
Figure S9. NOESY spectrum of **1** in $CDCl_3$.
Figure S10. (+)-HRCIMS spectrum of **2**.
Figure S11. Enlarged (+)-HRCIMS spectrum of **2**.
Figure S12. ^{1}H NMR spectrum of **2** in $CDCl_3$.
Figure S13. ^{13}C NMR spectrum of **2** in $CDCl_3$.
Figure S14. DEPT135 (upper) and DEPT90 (lower) spectra of **2** in $CDCl_3$.
Figure S15. HSQC spectrum of **2** in $CDCl_3$.
Figure S16. COSY spectrum of **2** in $CDCl_3$.
Figure S17. HMBC spectrum of **2** in $CDCl_3$.
Figure S18. NOESY spectrum of **2** in $CDCl_3$.
Figure S19. (+)-HRCIMS spectrum of **3**.
Figure S20. Enlarged (+)-HRCIMS spectrum of **3**.
Figure S21. ^{1}H NMR spectrum of **3** in $CDCl_3$.
Figure S22. ^{13}C NMR spectrum of **3** in $CDCl_3$.
Figure S23. ^{1}H NMR spectrum of **3** in acetone-d_6.
Figure S24. ^{13}C NMR spectrum of **3** in acetone-d_6.
Figure S25. DEPT135 (upper) and DEPT90 (lower) spectra of **3** in acetone-d_6.
Figure S26. HSQC spectrum of **3** in acetone-d_6.
Figure S27. COSY spectrum of **3** in acetone-d_6.
Figure S28. HMBC spectrum of **3** in acetone-d_6.
Figure S29. NOESY spectrum of **3** in acetone-d_6.
Figure S30. Experimental and GIAO NMR calculated data for 3*R**,4*S**,7*S**-**1** (isomer 1) and 3*R**,4*S**,7*R**-**1** (isomer 2) following the STS method. (a) Data were generated following the STS method without further dichloro correction. (b) Data were generated following the STS method with further correction for dichloro carbon (marked in red).
Figure S31. Experimental and GIAO NMR calculated data for 3*R**,4*S**,7*S**-**2** (isomer 1) and 3*R**,4*S**,7*R**-**2** (isomer 2) following the STS method. (a) Data were generated following the STS method without further dichloro correction. (b) Data were generated following the STS method with further correction for dichloro carbon (marked in red).
Figure S32. Experimental and GIAO NMR calculated data for 7*Z*-**3** (isomer 1) and 7*E*-**3** (isomer 2) following the STS method. (a) Data were generated following the STS method without further dibromo correction. (b) Data were generated following the STS

method with further correction for dibromo carbon (marked in red).

Table S1. ^{1}H and ^{13}C NMR spectroscopic data of **3**, **4**, and **5** in acetond-*d*$_{6}$.

Table S2. Comparison of specific optical rotations and selected NMR data of synthetic halogenated monoterpenes.

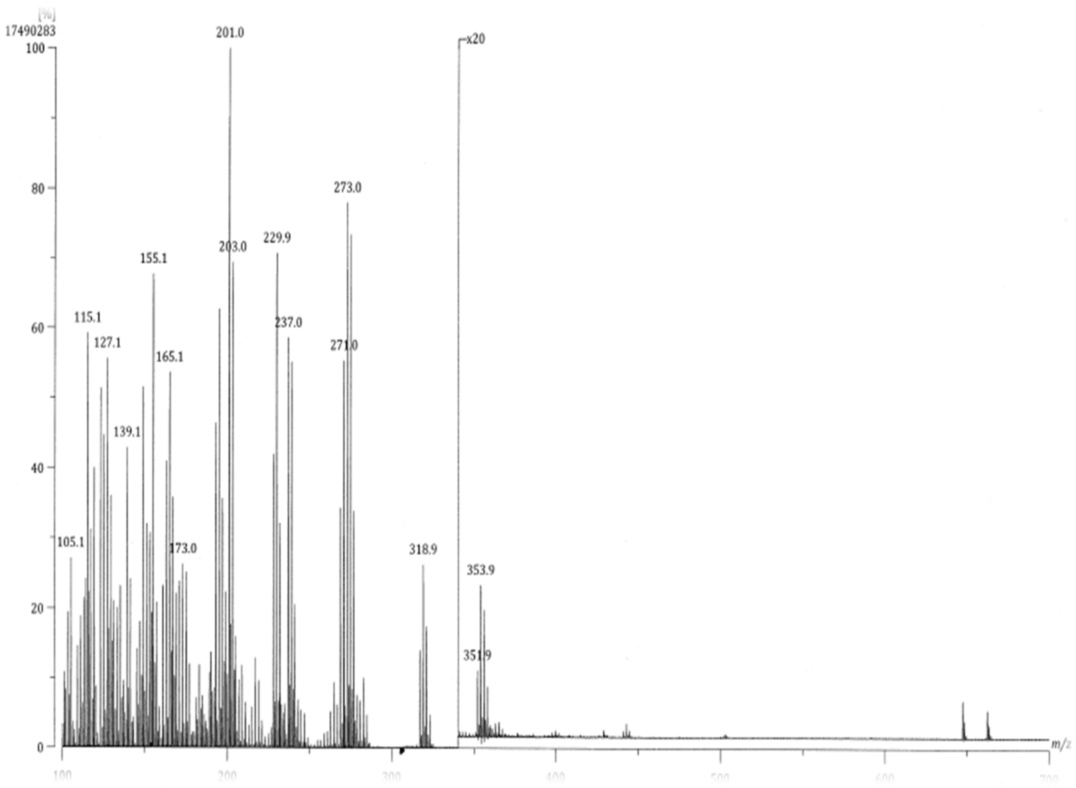

Figure S1. (+)-EIMS spectrum of **1**.

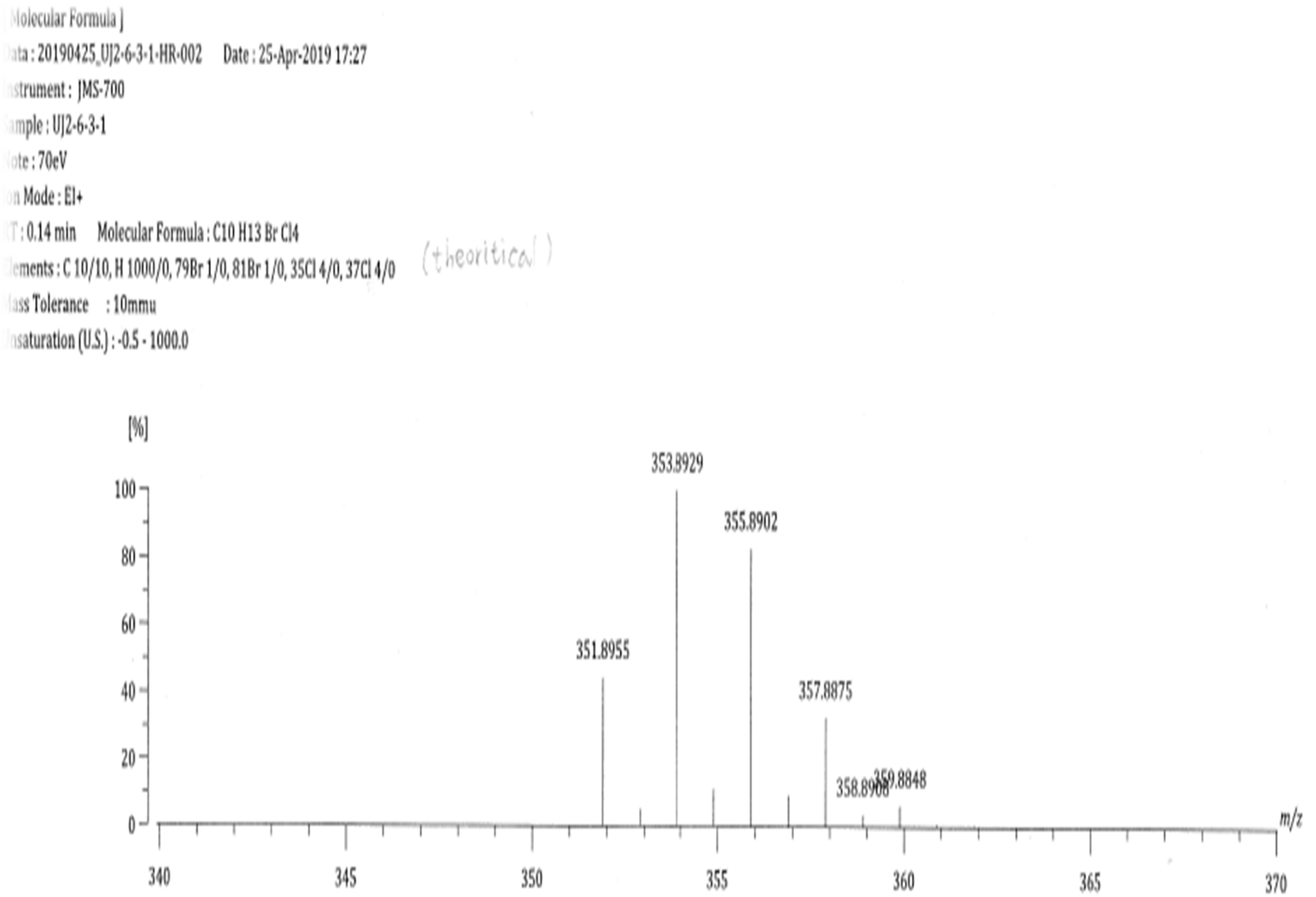

Figure S2. (+)-HREIMS spectrum of **1**.

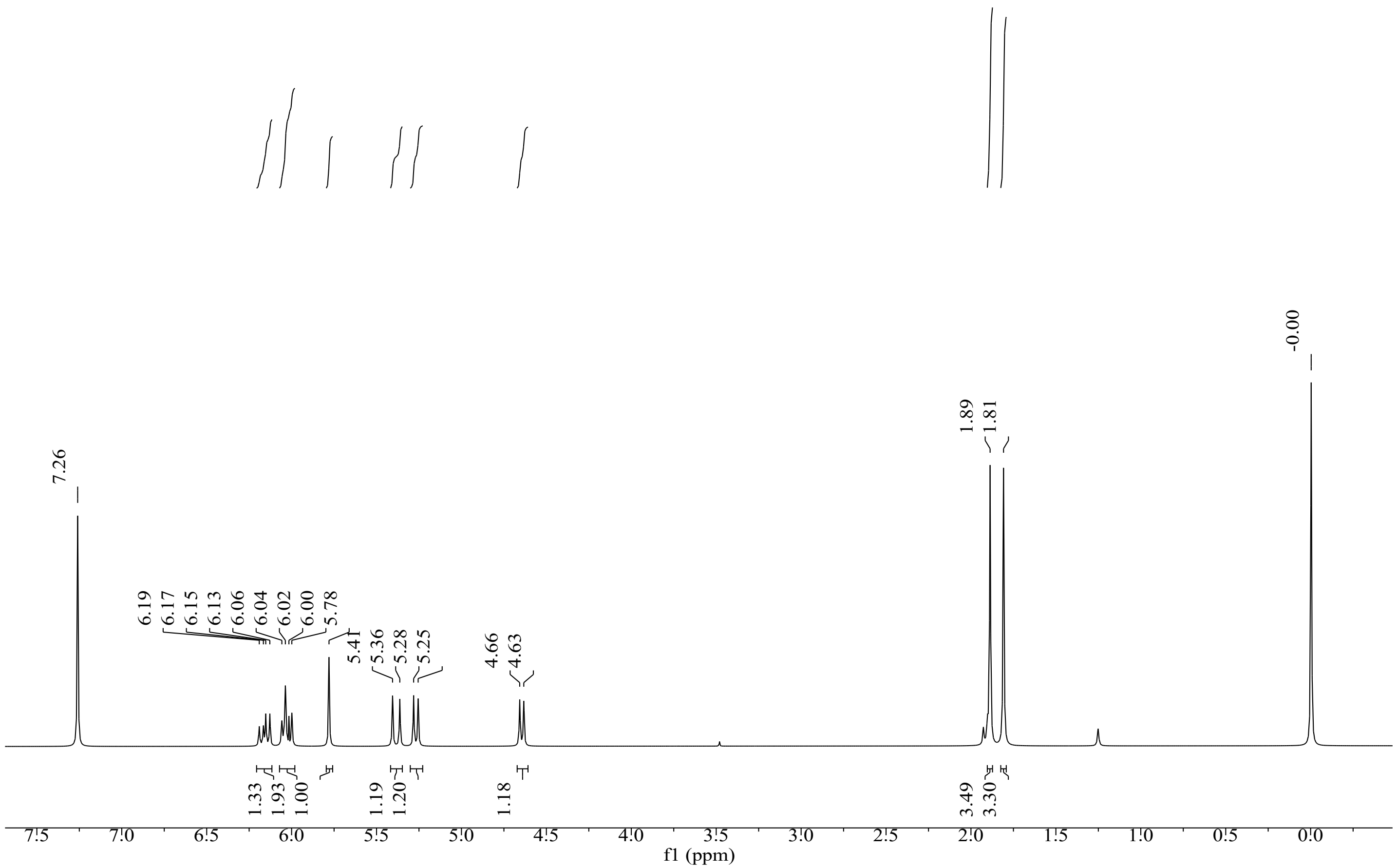

Figure S3. ^{1}H NMR spectrum of **1** in $CDCl_3$.

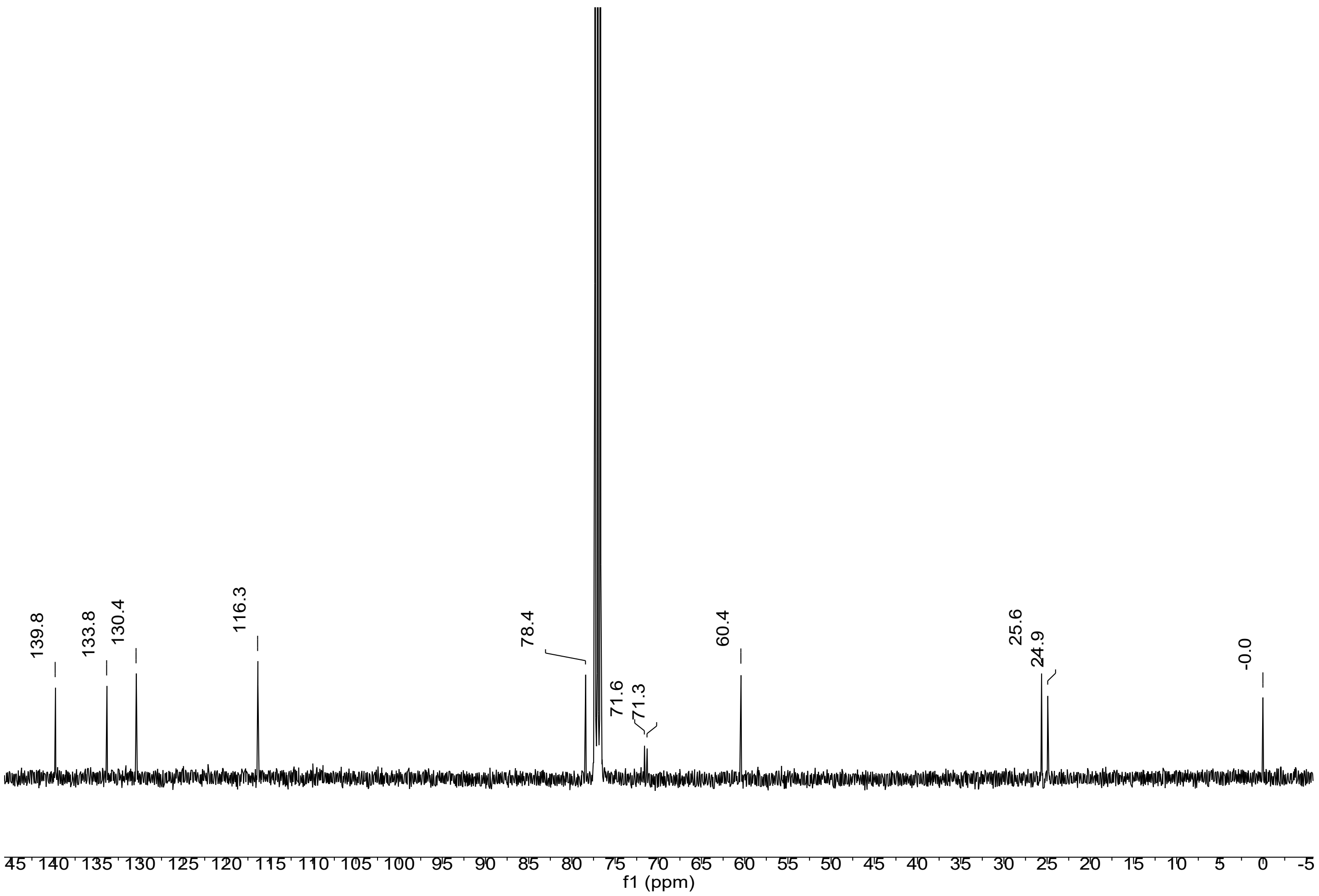

Figure S4. ^{13}C NMR spectrum of **1** in $CDCl_3$.

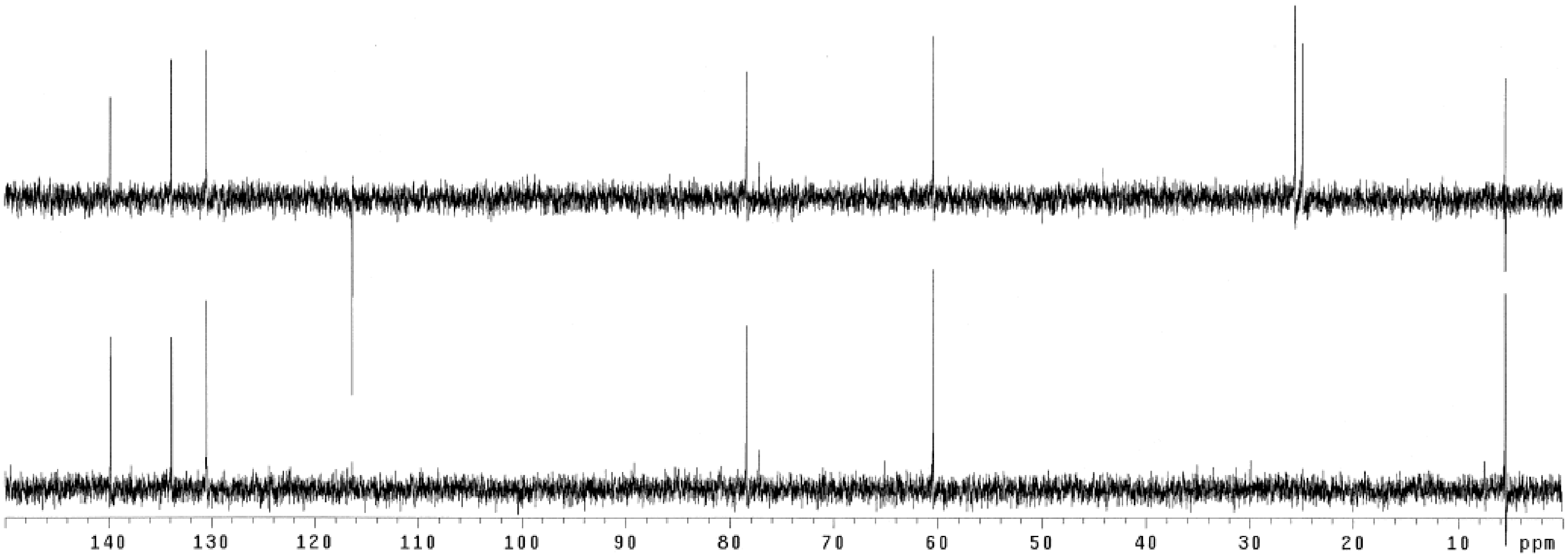

Figure S5. DEPT135 (upper) and DEPT90 (lower) spectra of **1** in $CDCl_3$.

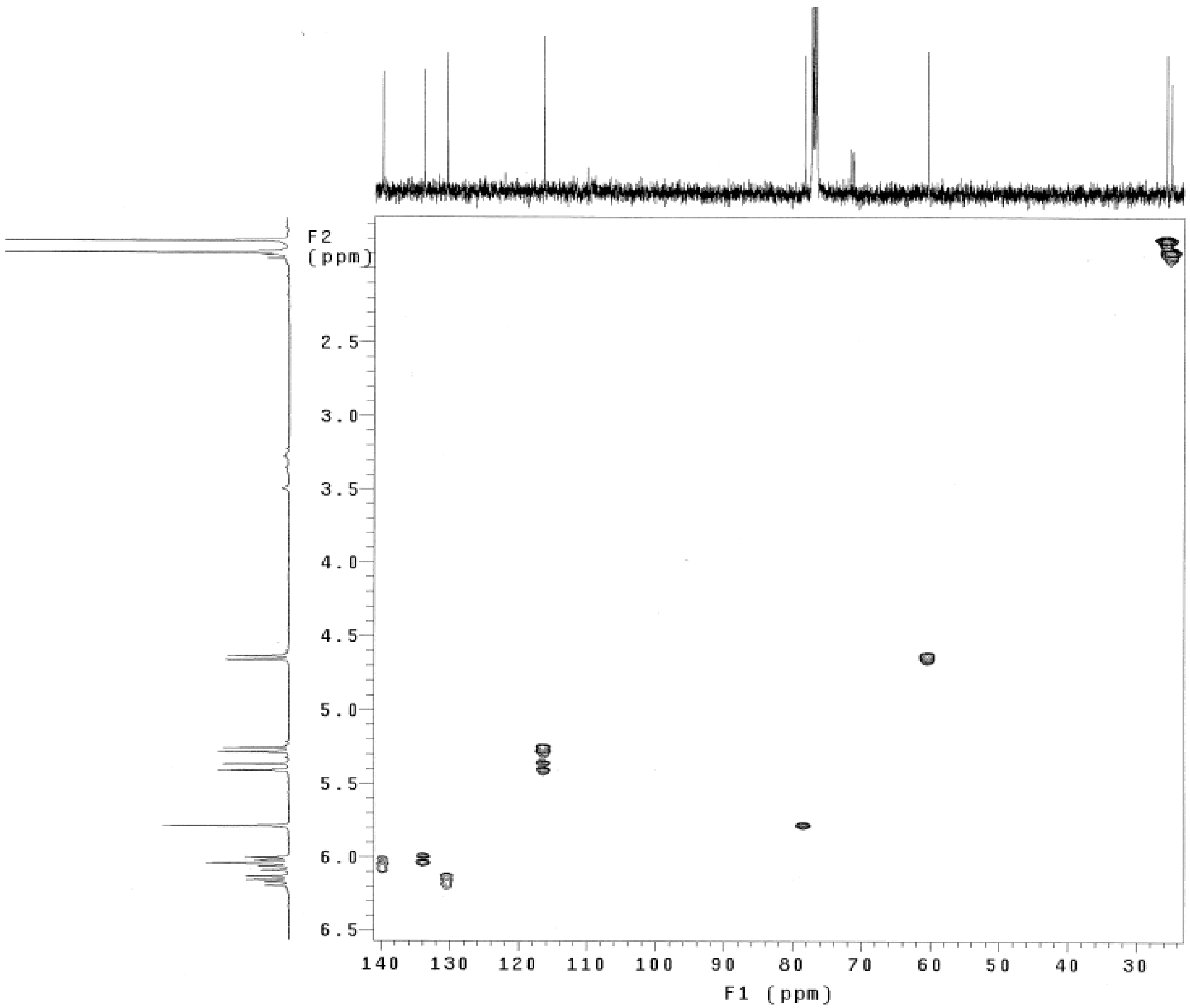

Figure S6. HSQC spectrum of **1** in $CDCl_3$.

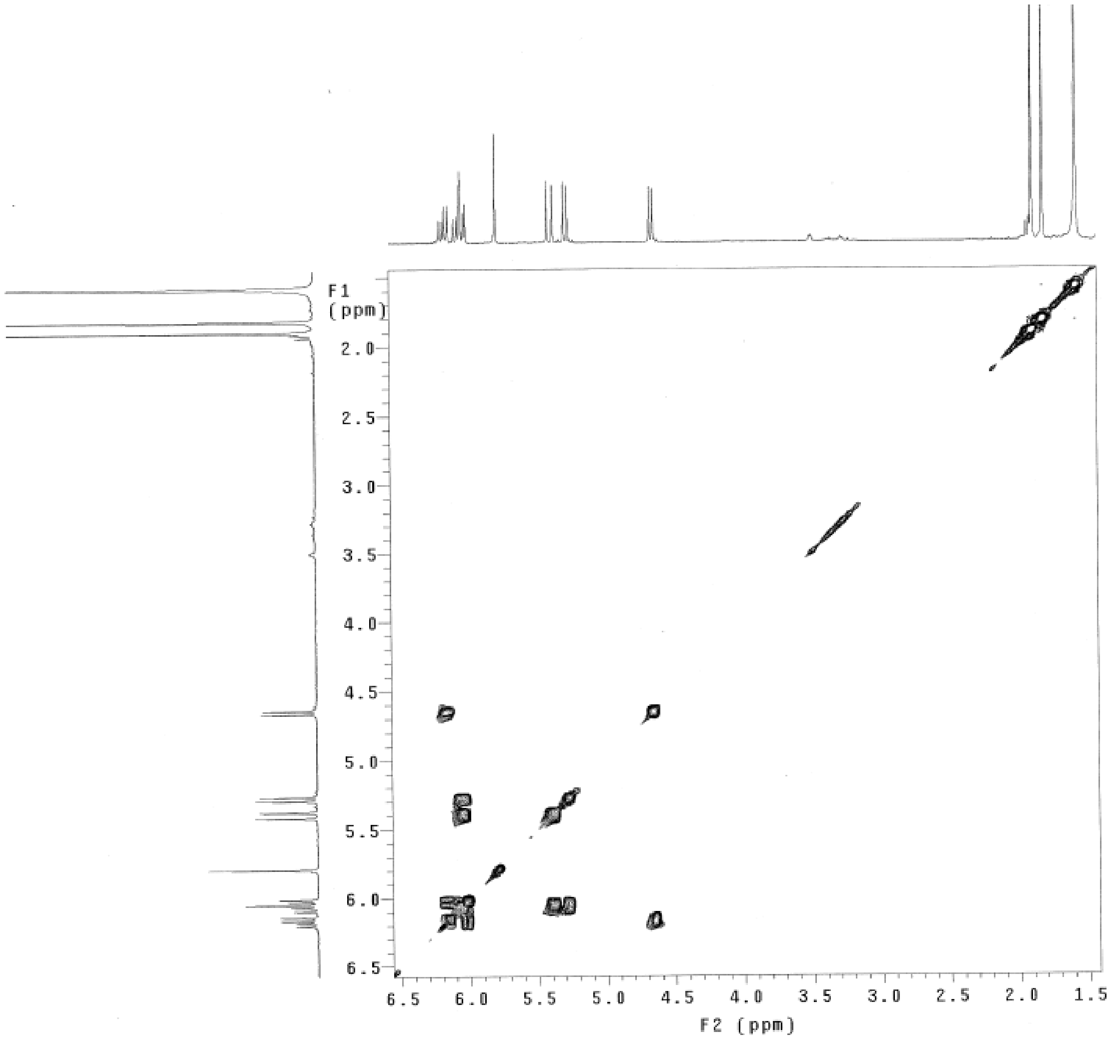

Figure S7. COSY spectrum of **1** in $CDCl_3$.

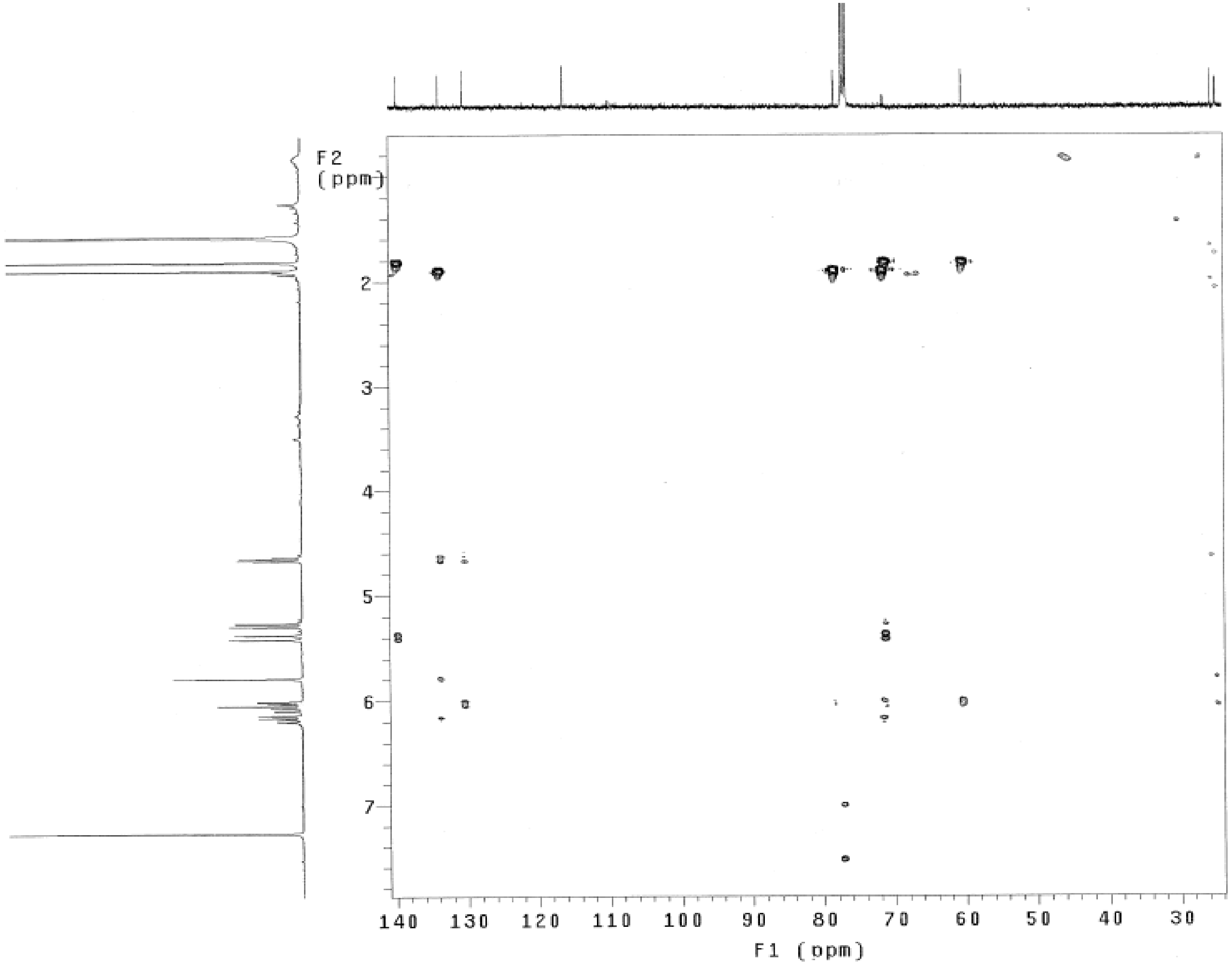

Figure S8. HMBC spectrum of **1** in $CDCl_3$.

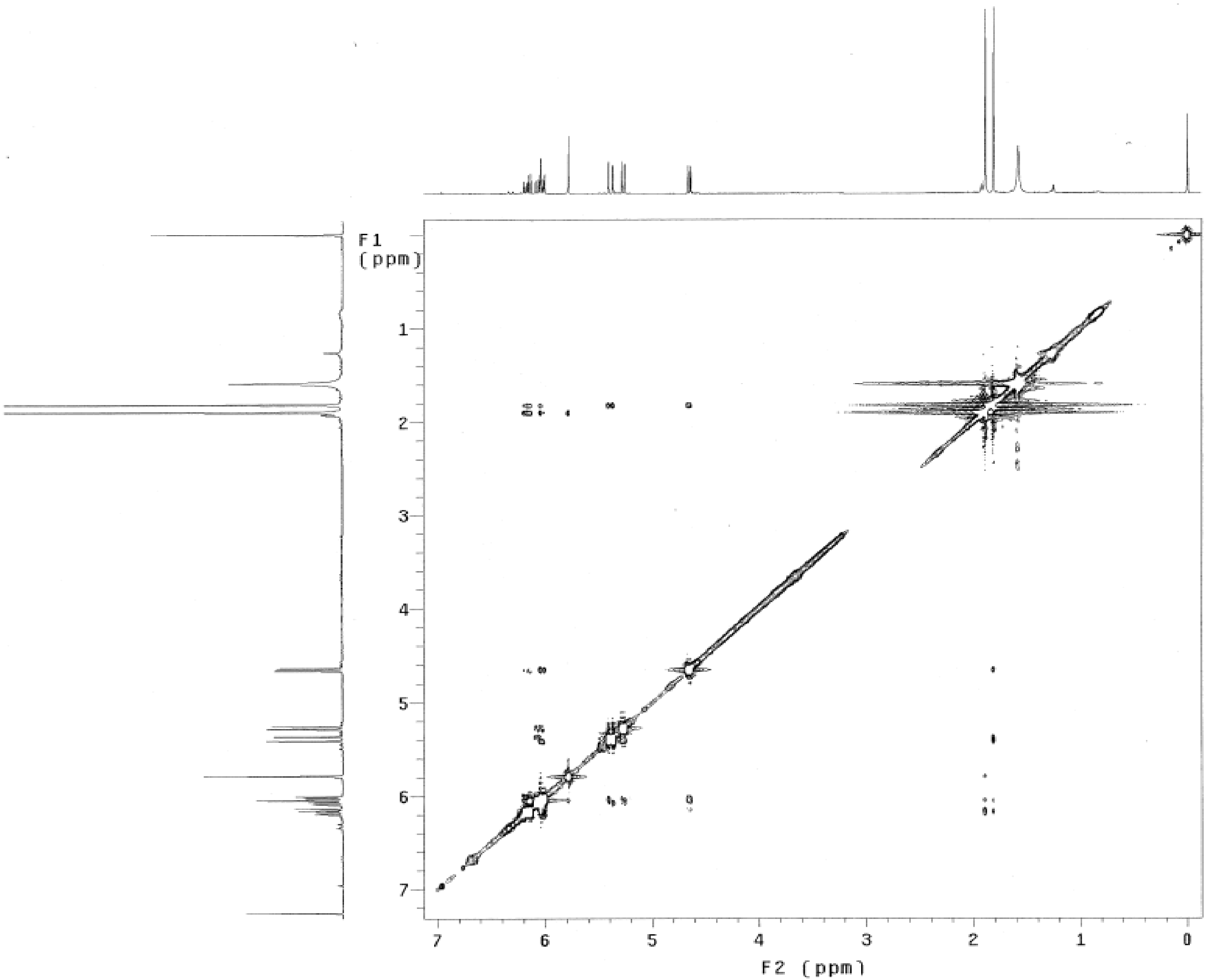

Figure S9. NOESY spectrum of **1** in $CDCl_3$.

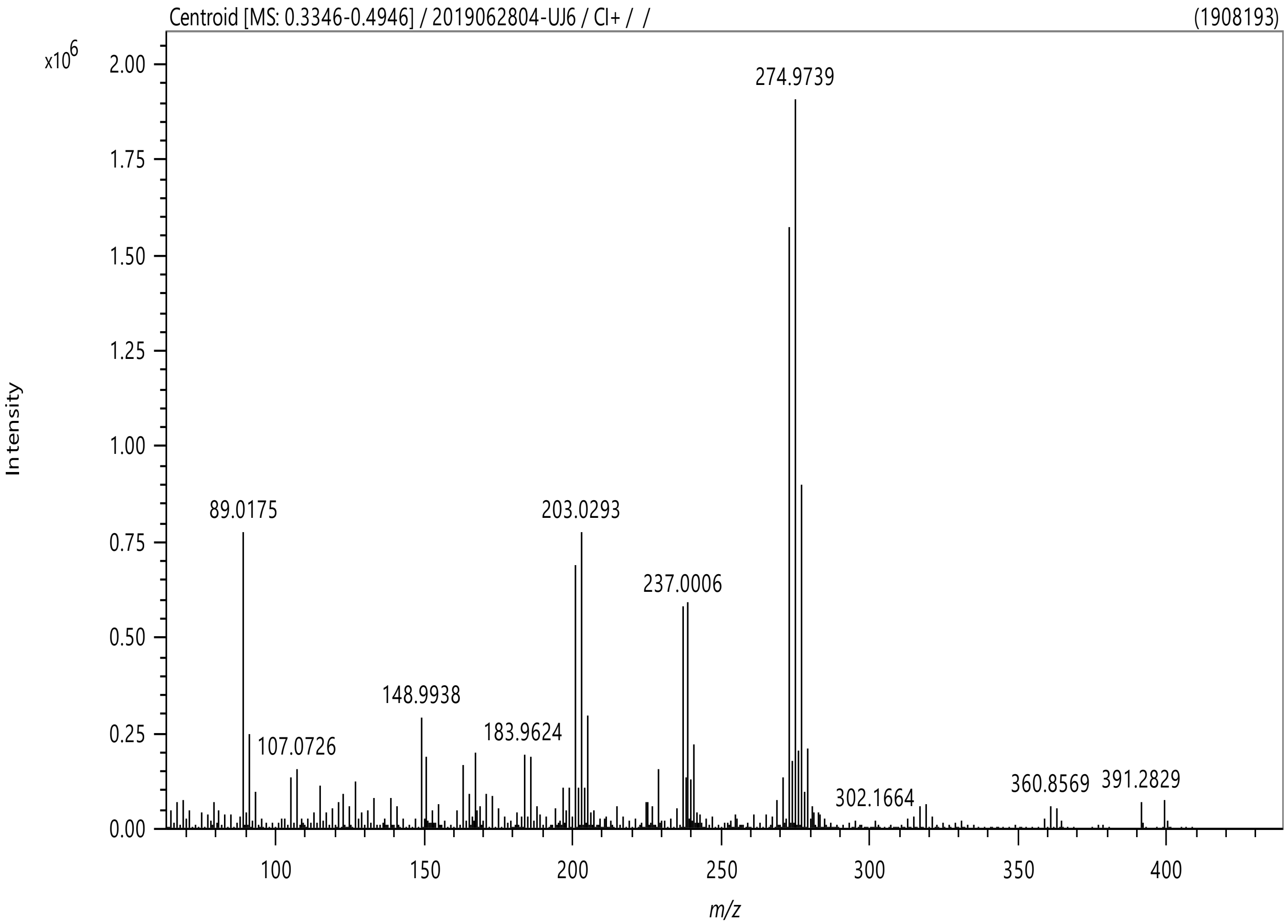

Figure S10. (+)-HRCIMS spectrum of **2**.

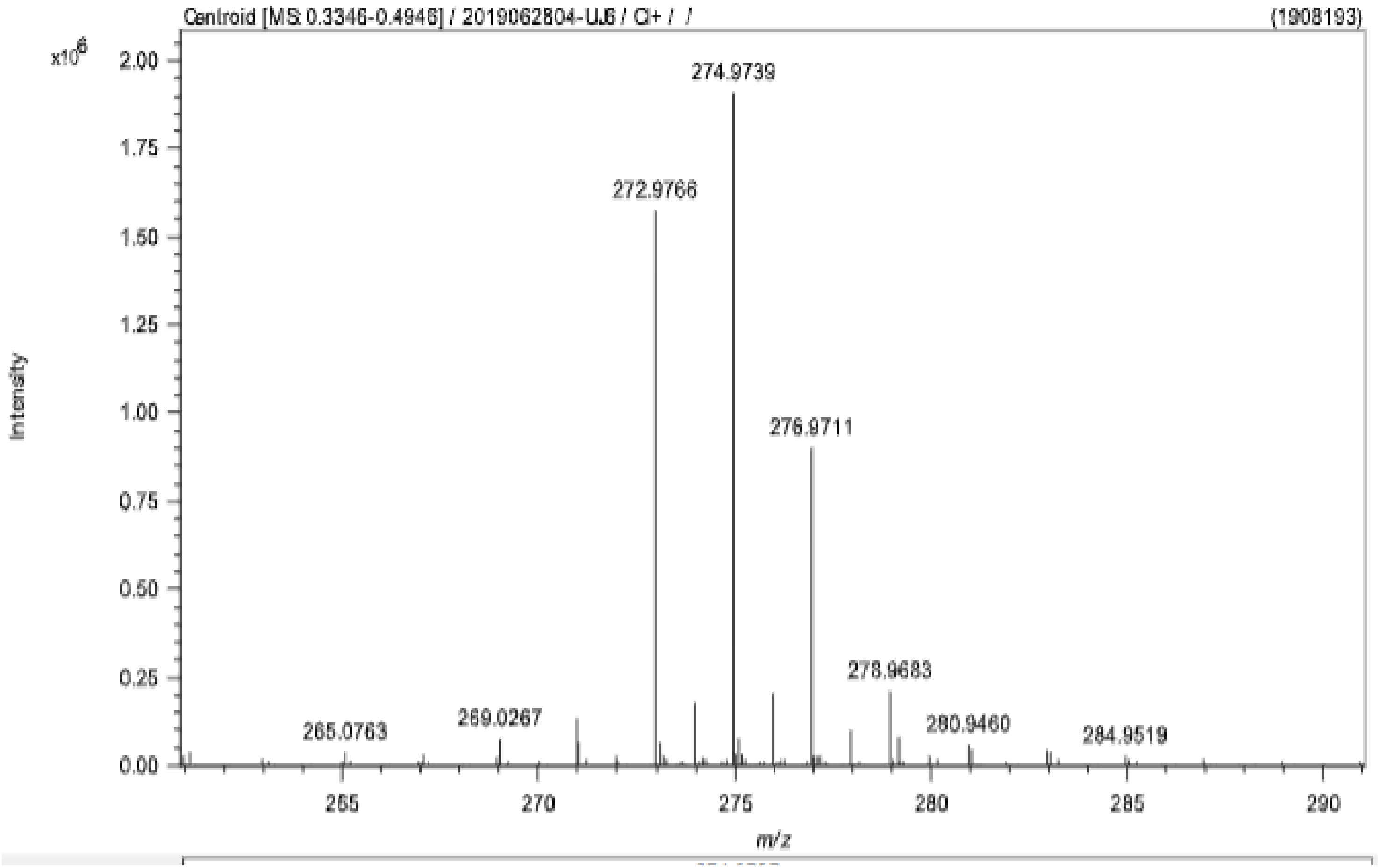

Figure S11. Enlarged (+)-HRCIMS spectrum of **2**.

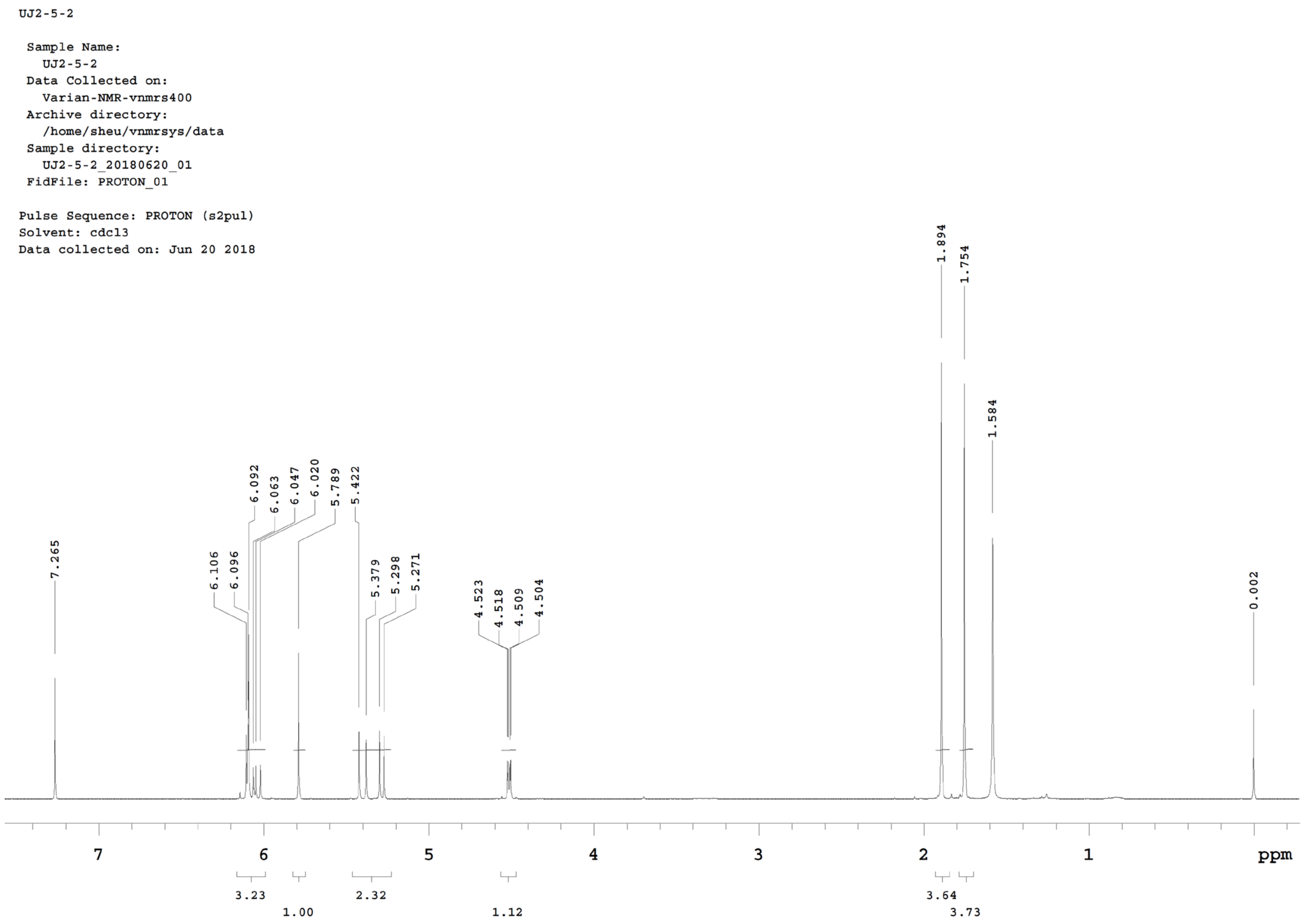

Figure S12. ^{1}H NMR spectrum of **2** in $CDCl_3$.

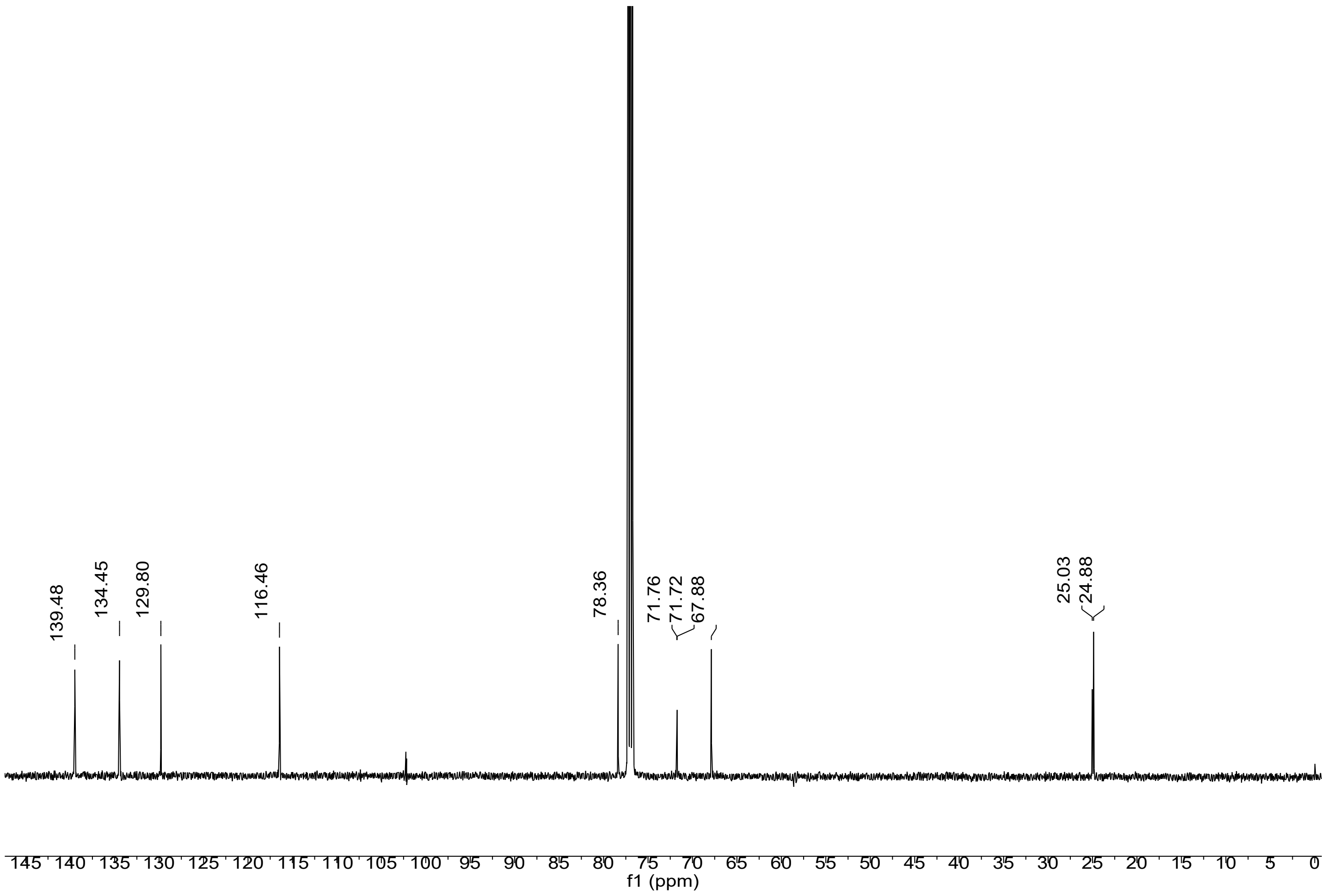

Figure S13. ^{13}C NMR spectrum of **2** in $CDCl_3$.

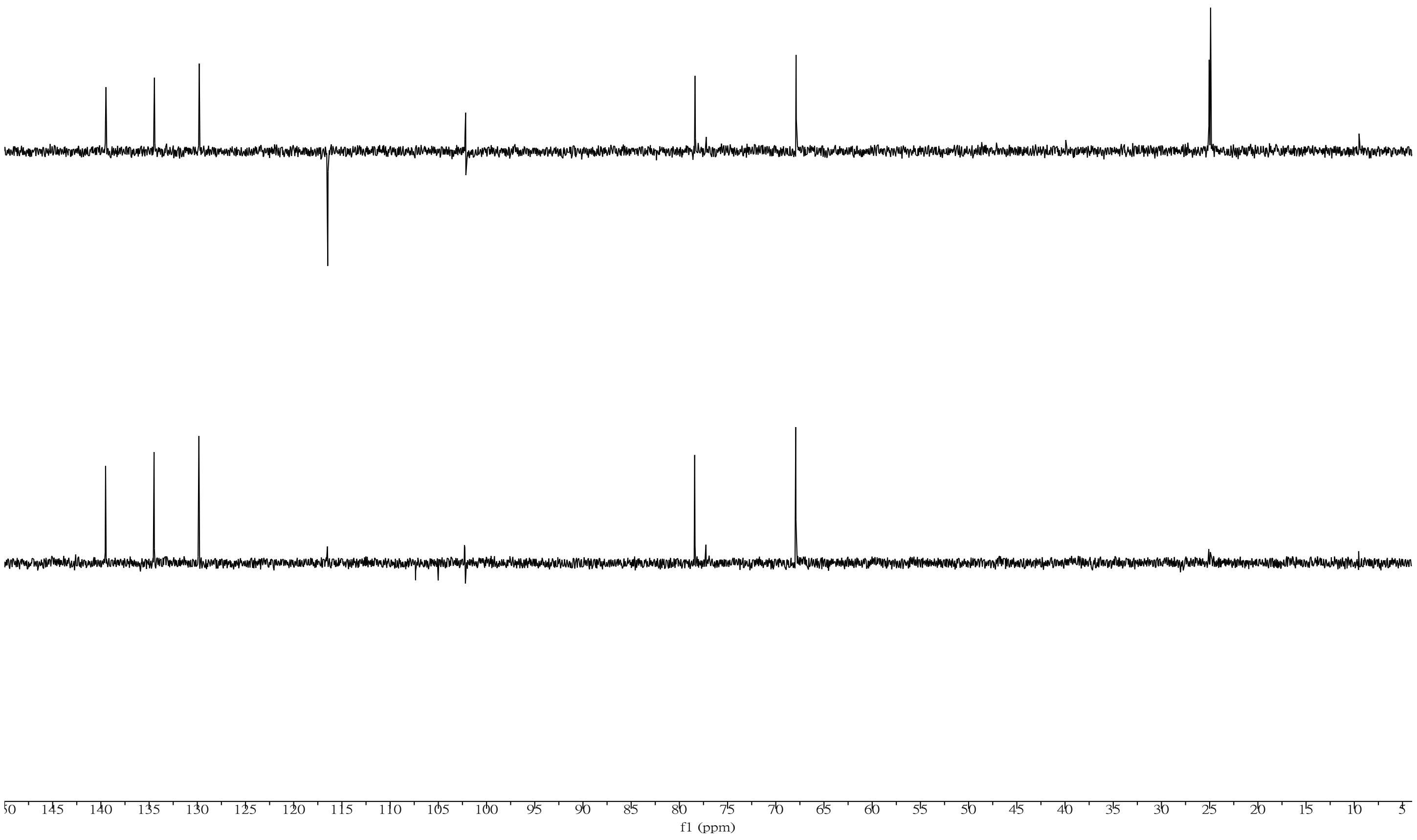

Figure S14. DEPT135 (upper) and DEPT90 (lower) spectra of **2** in $CDCl_3$.

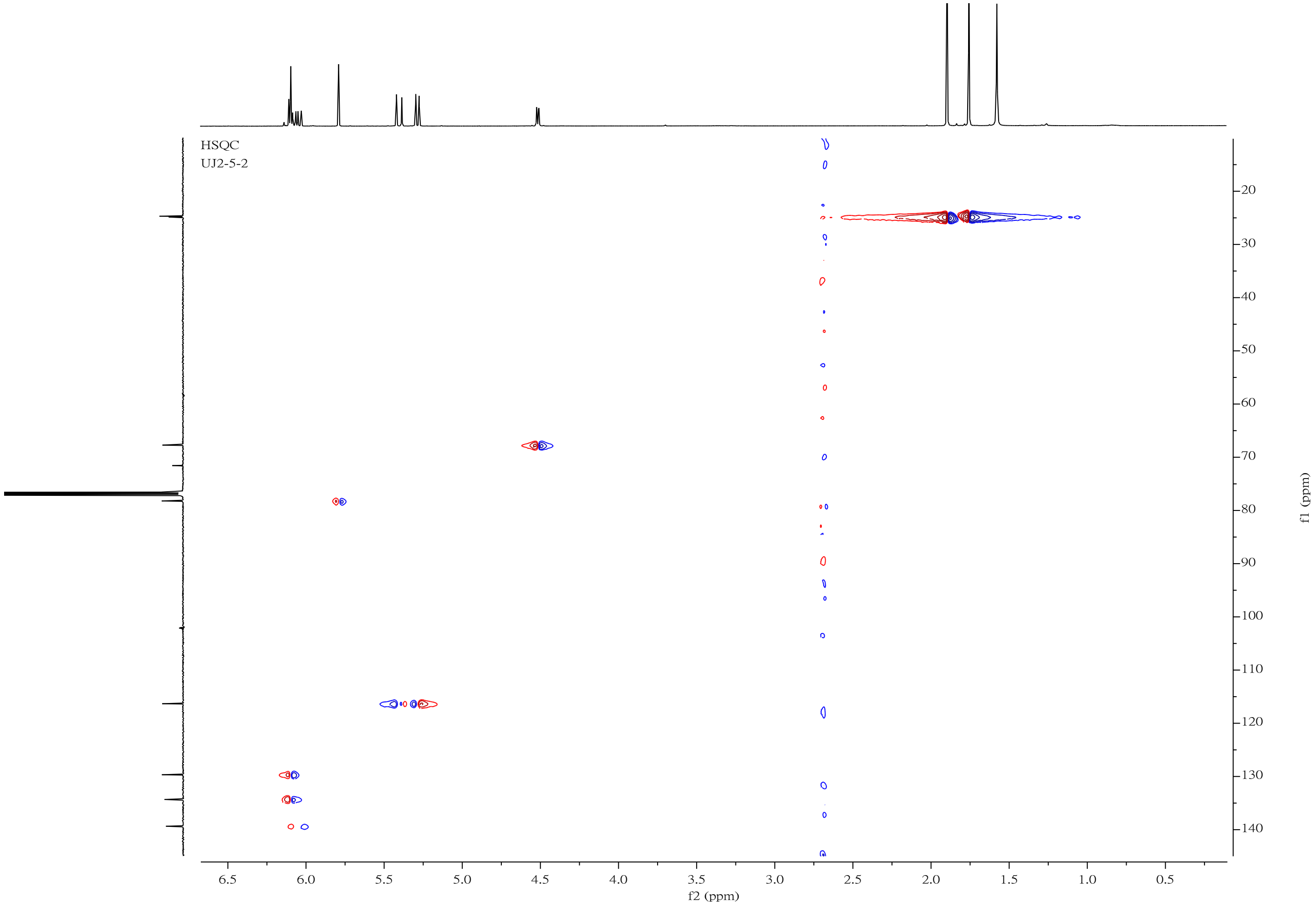

Figure S15. HSQC spectrum of **2** in $CDCl_3$.

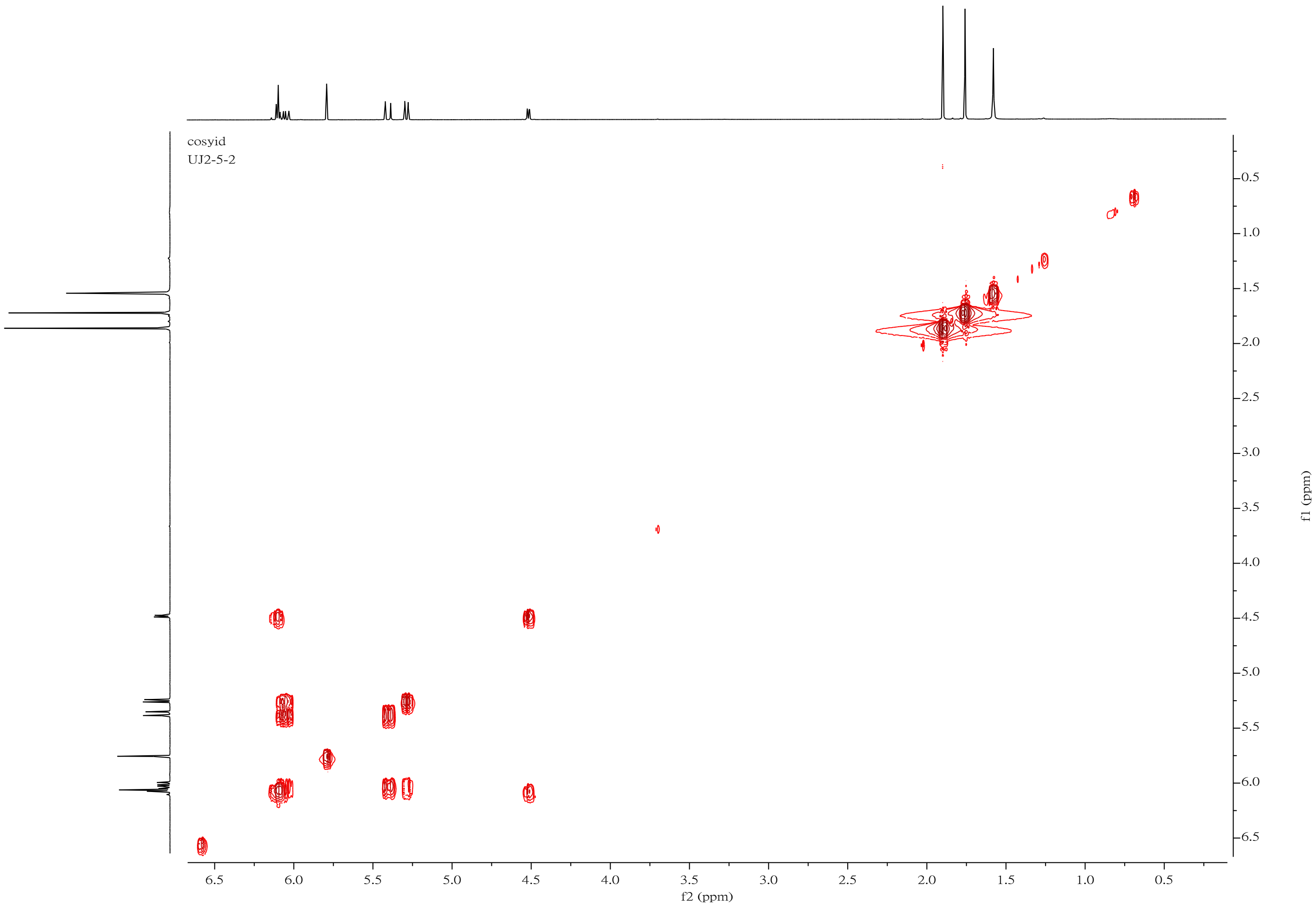

Figure S16. COSY spectrum of **2** in $CDCl_3$.

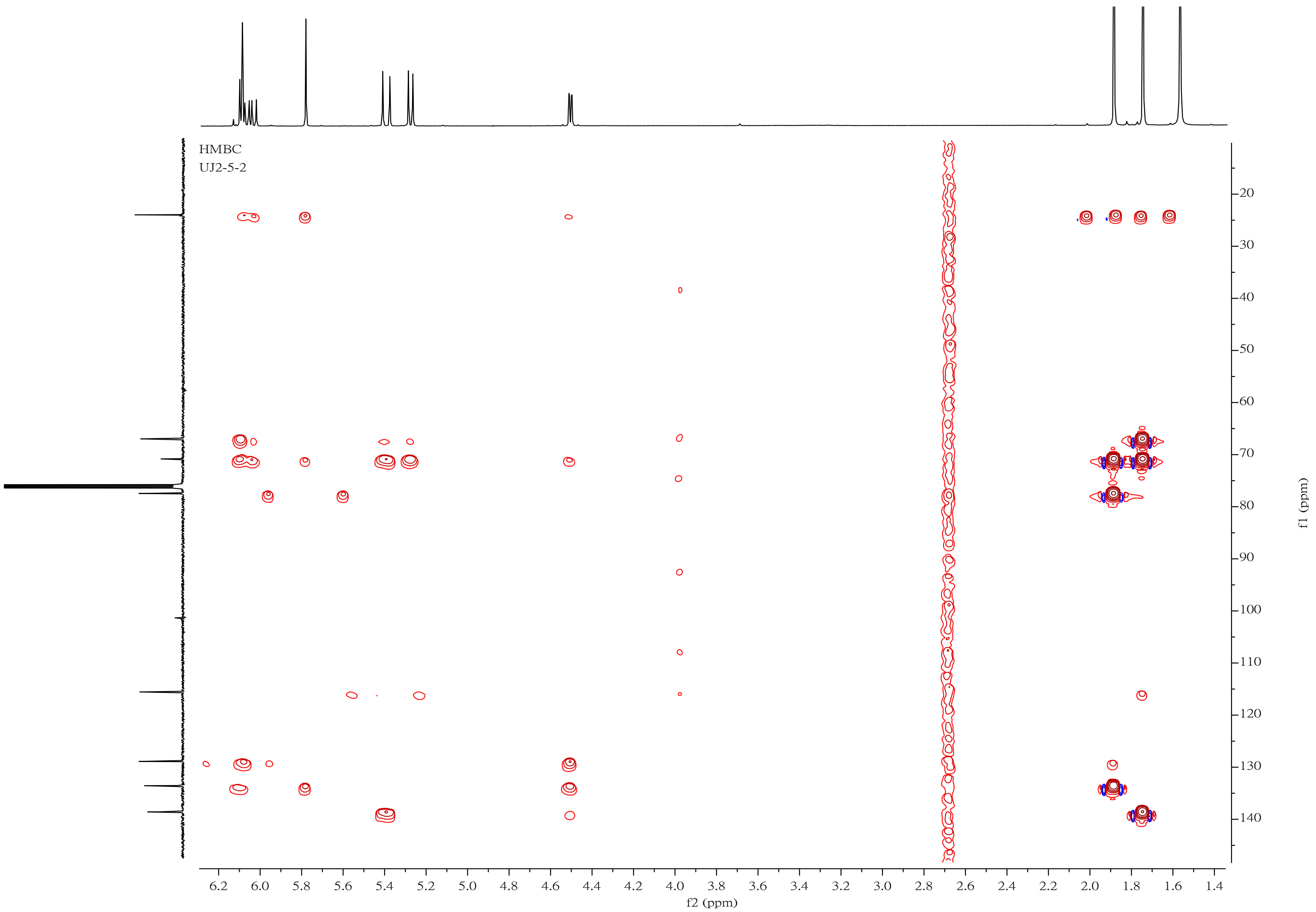

Figure S17. HMBC spectrum of **2** in $CDCl_3$.

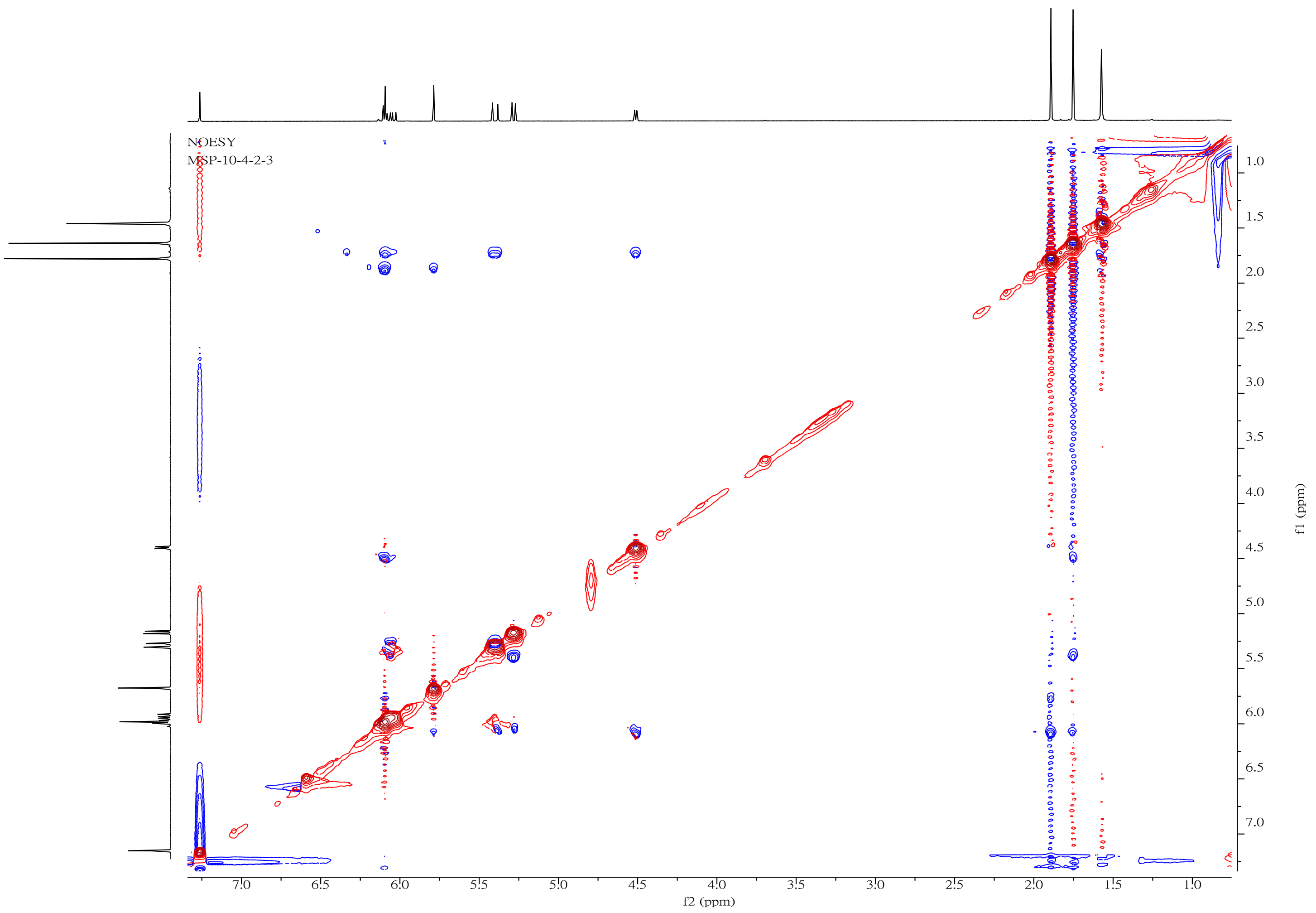

Figure S18. NOESY spectrum of **2** in $CDCl_3$.

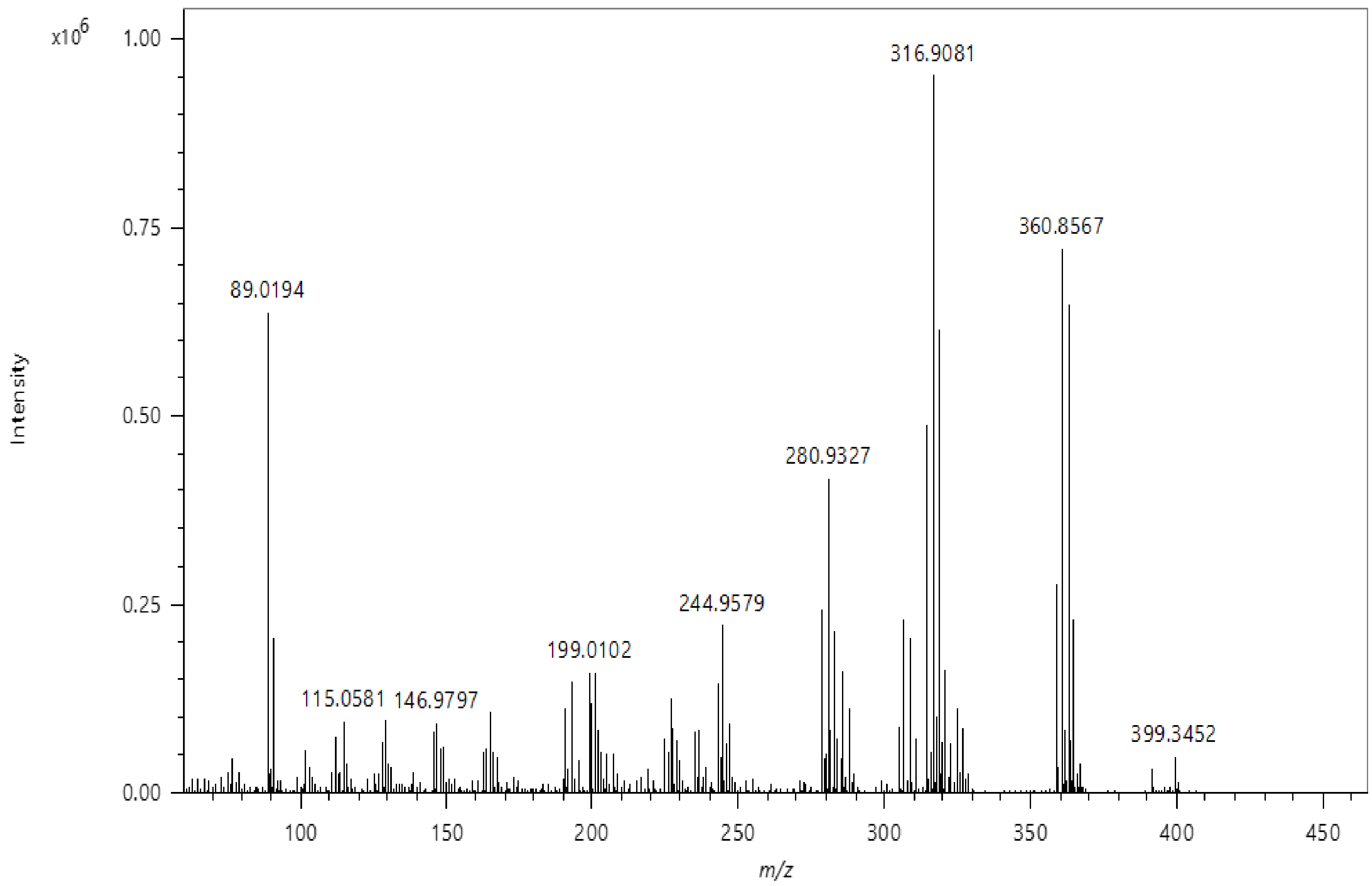

Figure S19. (+)-HRCIMS spectrum of **3**.

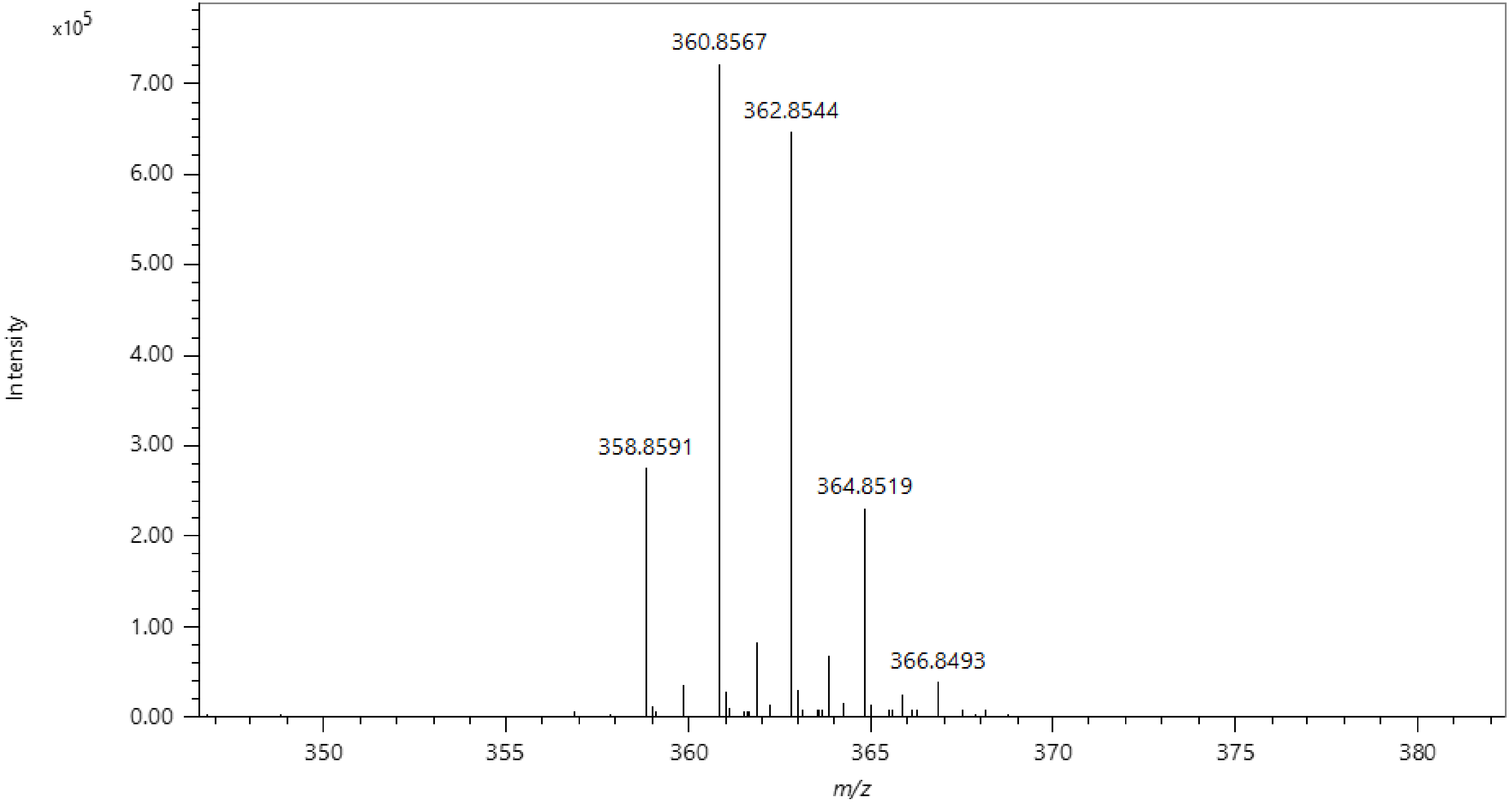

Figure S20. Enlarged (+)-HRCIMS spectrum of **3**.

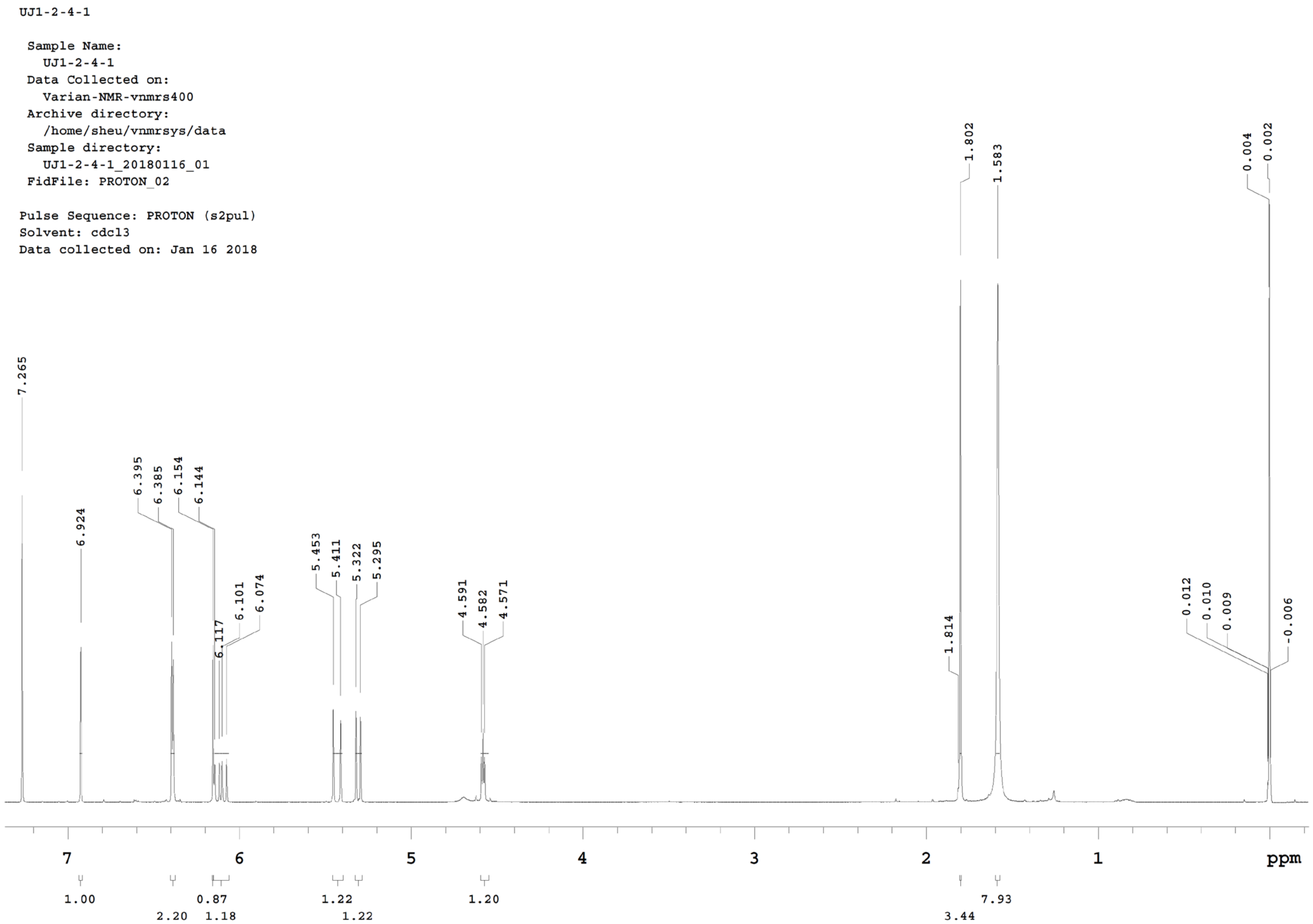

Figure S21. ^{1}H NMR spectrum of **3** in $CDCl_3$.

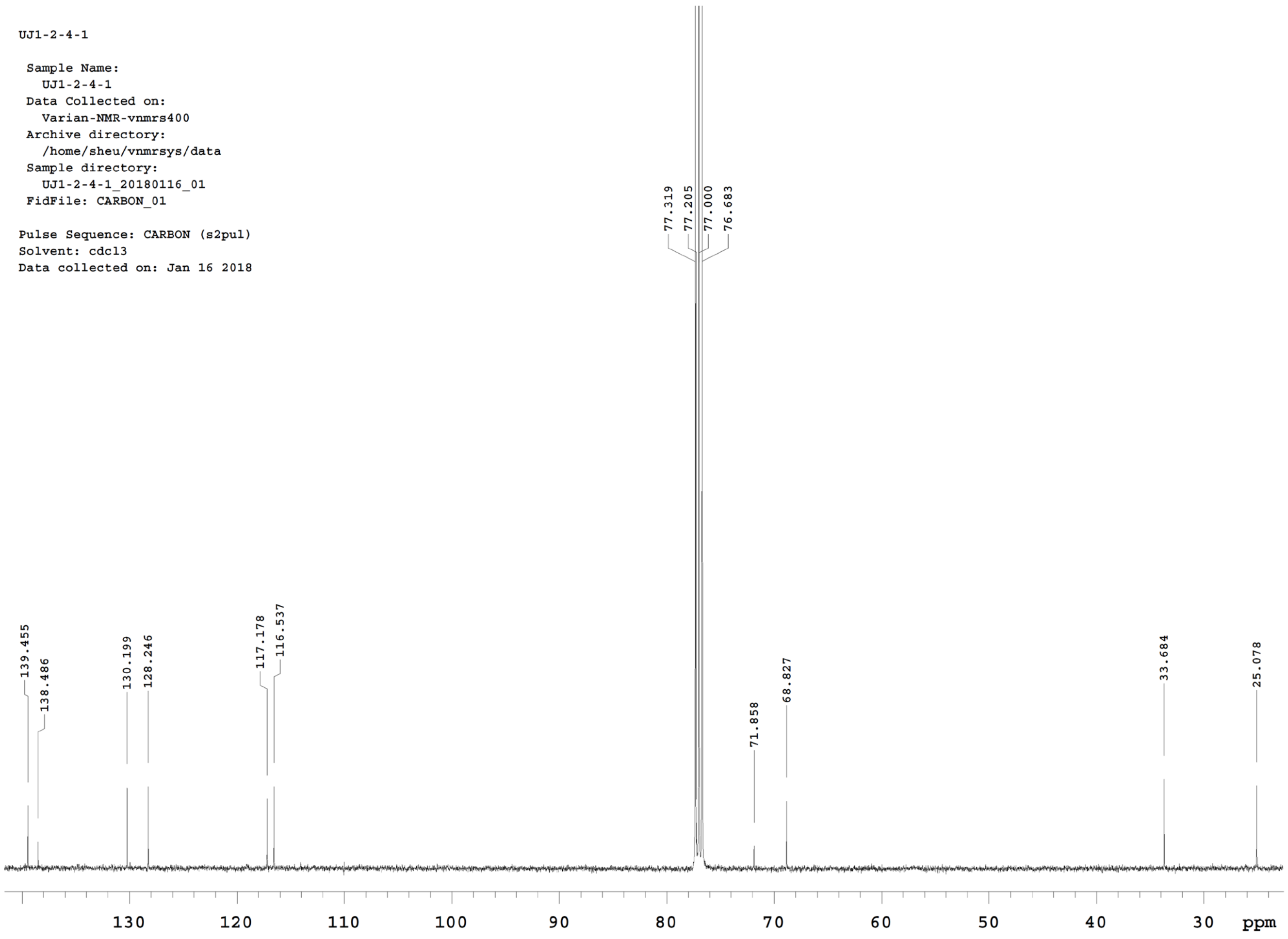

Figure S22. ^{13}C NMR spectrum of **3** in $CDCl_3$.

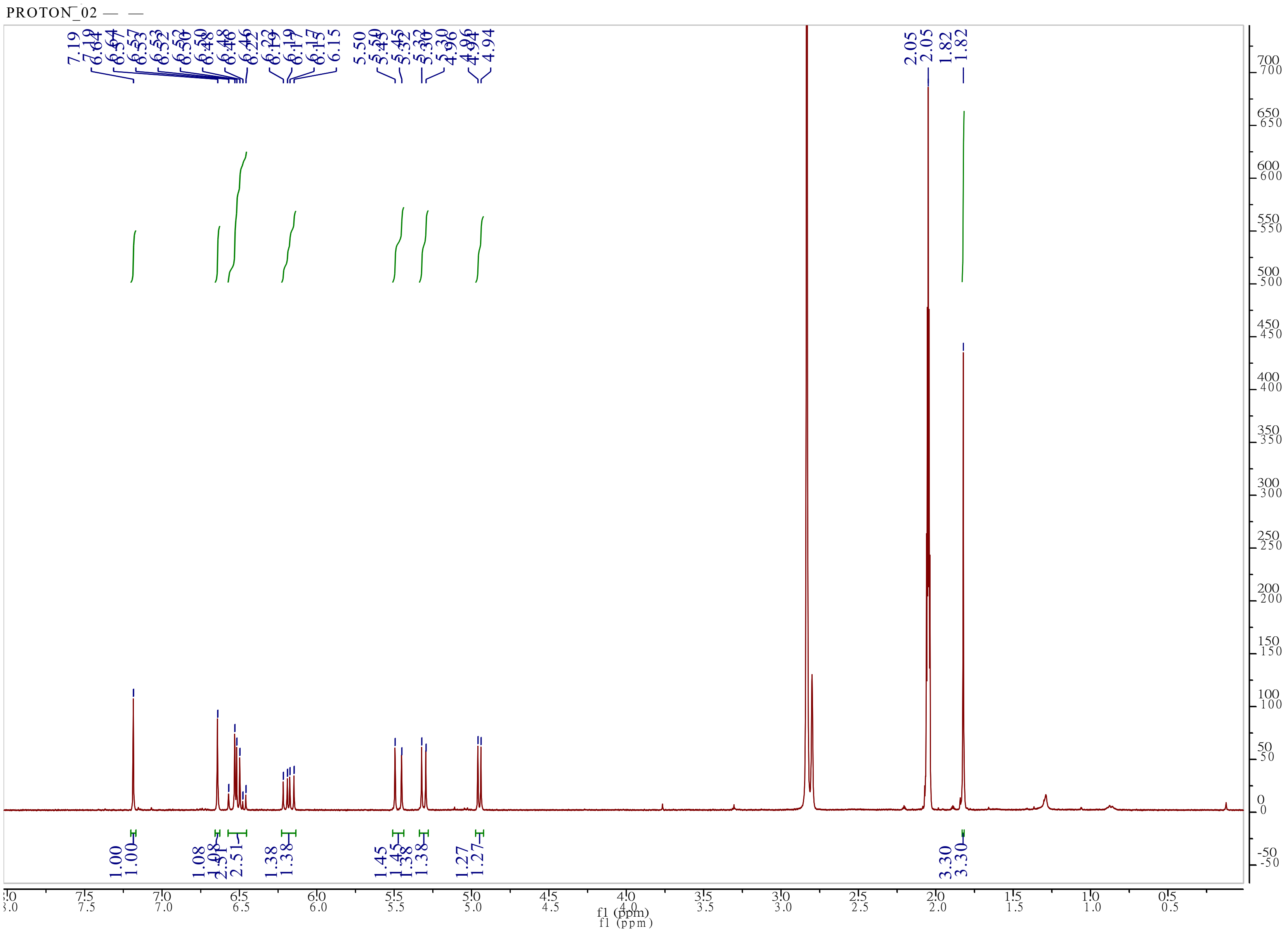

Figure S23. ^{1}H NMR spectrum of **3** in acetone-*d*₆.

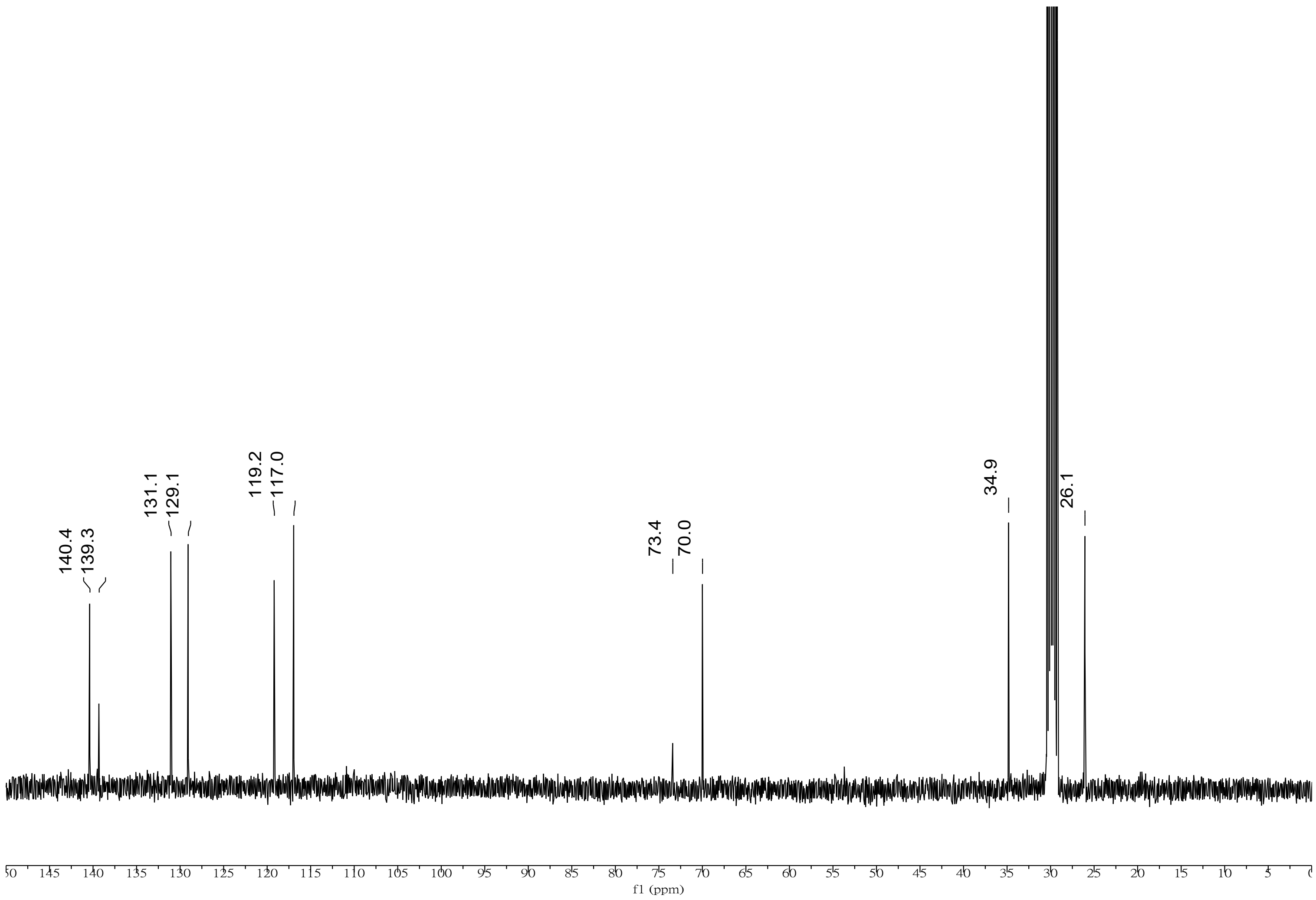

Figure S24. ^{13}C NMR spectrum of **3** in acetone-d_6.

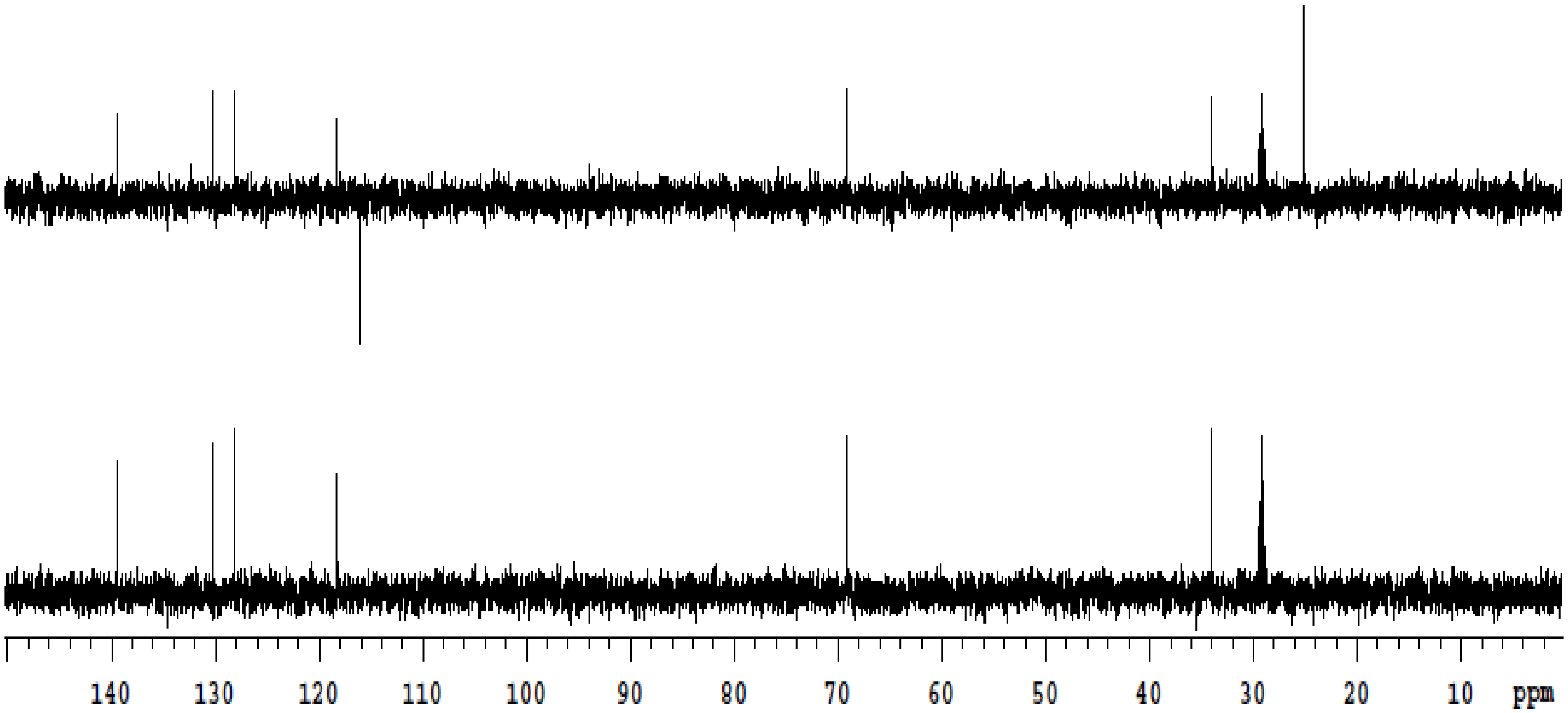

Figure S25. DEPT135 (upper) and DEPT90 (lower) spectra of **3** in acetone-d_6.

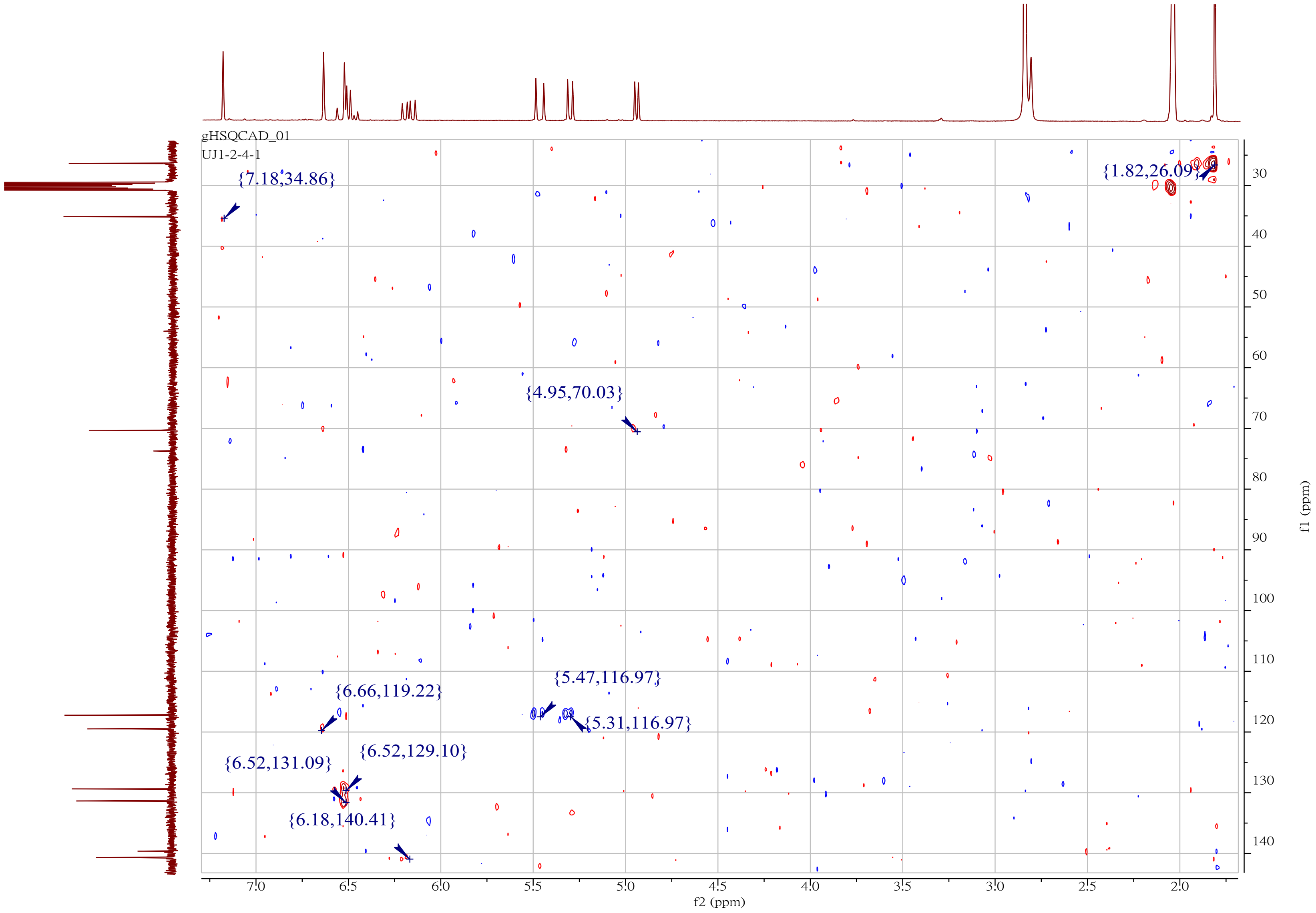

Figure S26. HSQC spectrum of **3** in acetone-d_6.

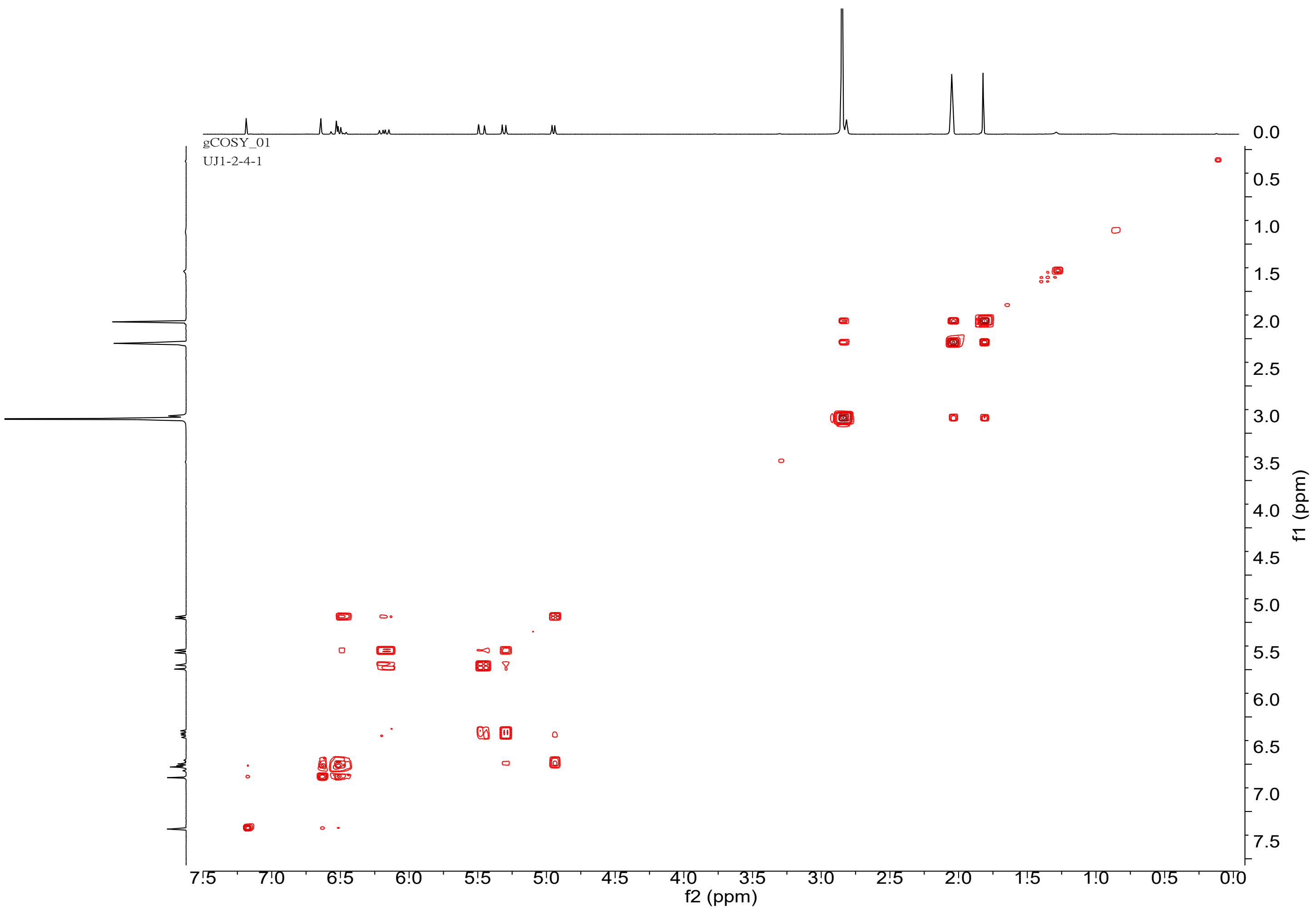

Figure S27. COSY spectrum of **3** in acetone-d_6.

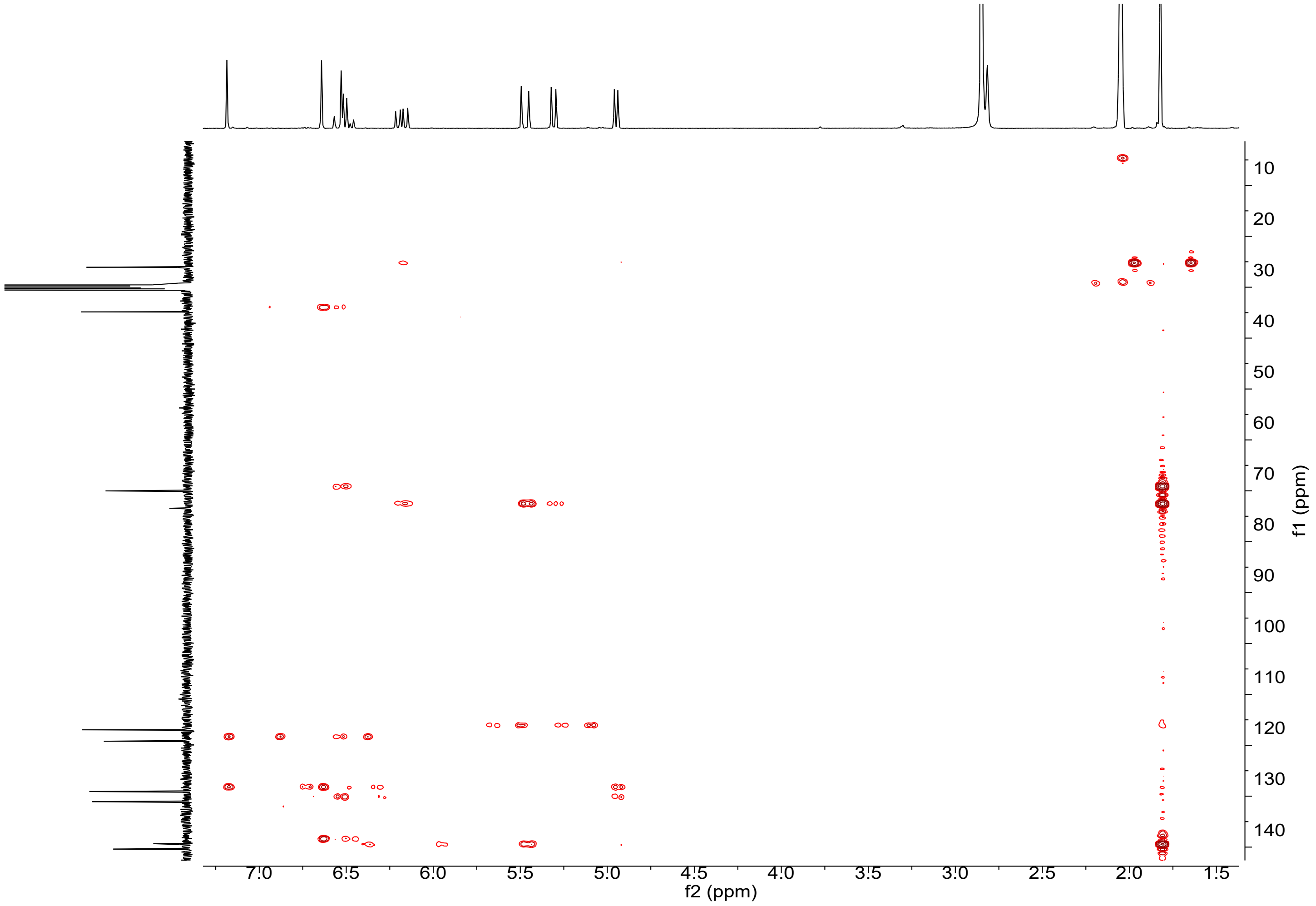

Figure S28. HMBC spectrum of **3** in acetone-d_6.

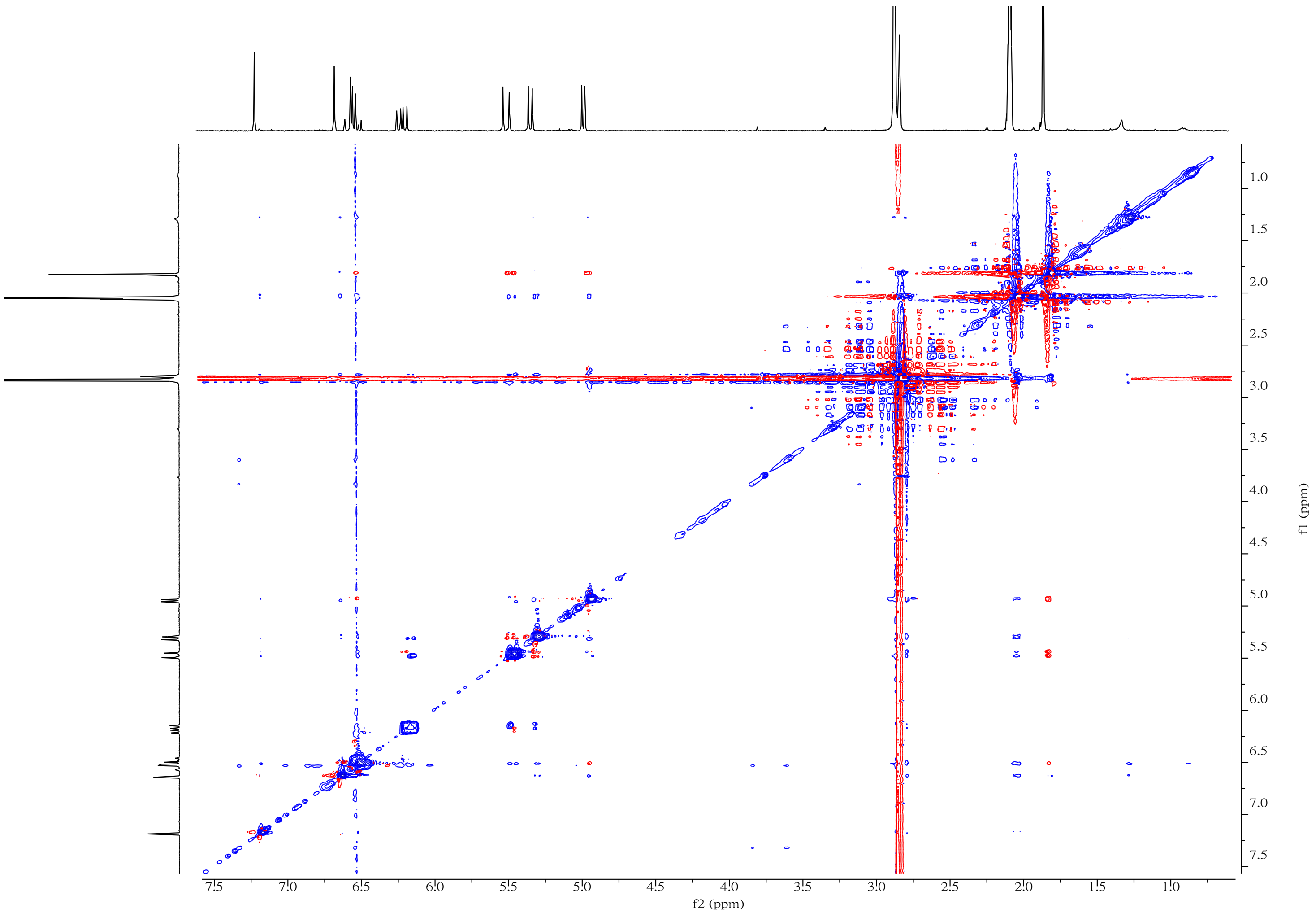

Figure S29. NOESY spectrum of **3** in acetone-d_6.

(a)

Note:
Pmean > 5% is the confidence interval

Note:
sp carbons include C≡N
sp2-CH/C for double bonds except carbonyl group
C=O includes only ketones and aldehydes

	Isomer 1	Isomer 2
MAE	1.27	1.27
RMS	1.51	1.50
P_{mean}	21.71%	22.28%
Prel	43.55%	56.45%

Heavy atom beari	Type of carbon	Carbon no.	Exptl δ	Isomer 1 Shielding tens	Corr Shielding	Calcd δ	Calcd δ (S(	abs dev	P	Isomer 2 Shielding tens	Corr Shielding	Calcd δ	Calcd δ (S(	abs dev	P	S
C-Cl	sp3-CH2/CH/C		78.4	106.403762	115.7754745	80.95	81.33	2.93	0.01	106.591796	115.95	80.78	81.11	2.71	0.01	
C-Cl	sp3-CH2/CH/C		71.6	116.2302597	124.6409403	71.85	72.25	0.65	0.56	116.0448467	124.47	72.03	72.37	0.77	0.49	
	sp2-CH		133.8	66.10566204	66.10566204	132.71	133.01	0.79	0.61	65.93316549	65.93	132.87	133.11	0.69	0.65	
	sp2-CH		130.4	68.24832935	68.24832935	130.71	131.02	0.62	0.69	68.11119355	68.11	130.84	131.08	0.68	0.66	
C-Br	sp3-CH2/CH/C		60.4	123.9684263	137.8868988	58.26	58.67	1.73	0.12	123.9459762	137.86	58.28	58.65	1.75	0.11	
C-Cl	sp3-CH2/CH/C		71.3	114.9072949	123.4473614	73.08	73.47	2.17	0.05	114.8281229	123.38	73.15	73.49	2.19	0.05	
	sp2-CH		139.8	60.74881305	60.74881305	137.70	138.00	1.80	0.24	60.80547012	60.81	137.65	137.88	1.92	0.21	
	sp2-CH2		116.4	80.70268373	80.70268373	115.85	116.17	0.23	0.60	80.60929049	80.61	115.94	116.20	0.20	0.65	
	sp3-CH3		24.9	171.4031183	171.4031183	23.83	24.29	0.61	0.56	171.1996226	171.20	24.04	24.47	0.43	0.68	
	sp3-CH3		25.6	171.307225	171.307225	23.93	24.39	1.21	0.24	171.4205303	171.42	23.81	24.24	1.36	0.19	

(b)

Note:
Pmean > 5% is the confidence interval

Note:
sp carbons include C≡N
sp2-CH/C for double bonds except carbonyl group
C=O includes only ketones and aldehydes

	Isomer 1	Isomer 2
MAE	1.07	1.16
RMS	1.33	1.41
P_{mean}	28.75%	25.43%
Prel	77.33%	22.67%

Heavy atom beari	Type of carbon	Carbon no.	Exptl δ	Isomer 1 Shielding tens	Corr Shielding	Calcd δ	Calcd δ (S(	abs dev	P	Isomer 2 Shielding tens	Corr Shielding	Calcd δ	Calcd δ (S(	abs dev	P	S
None	sp3-CH2/CH/C		78.4	121.1445606	121.1445606	75.44	76.40	2.00	0.07	121.306514	121.31	75.28	76.18	2.22	0.05	
C-Cl	sp3-CH2/CH/C		71.6	116.2302597	124.6409403	71.85	72.82	1.22	0.27	116.0448467	124.47	72.03	72.94	1.34	0.23	
	sp2-CH		133.8	66.10566204	66.10566204	132.71	133.47	0.33	0.83	65.93316549	65.93	132.87	133.56	0.24	0.87	
	sp2-CH		130.4	68.24832935	68.24832935	130.71	131.48	1.08	0.48	68.11119355	68.11	130.84	131.53	1.13	0.46	
C-Br	sp3-CH2/CH/C		60.4	123.9684263	137.8868988	58.26	59.27	1.13	0.31	123.9459762	137.86	58.28	59.25	1.15	0.30	
C-Cl	sp3-CH2/CH/C		71.3	114.9072949	123.4473614	73.08	74.04	2.74	0.01	114.8281229	123.38	73.15	74.07	2.77	0.01	
	sp2-CH		139.8	60.74881305	60.74881305	137.70	138.44	1.36	0.38	60.80547012	60.81	137.65	138.32	1.48	0.34	
	sp2-CH2		116.4	80.70268373	80.70268373	115.85	116.66	0.26	0.54	80.60929049	80.61	115.94	116.69	0.29	0.51	
	sp3-CH3		24.9	171.4031183	171.4031183	23.83	24.96	0.06	0.95	171.1996226	171.20	24.04	25.15	0.25	0.81	
	sp3-CH3		25.6	171.307225	171.307225	23.93	25.06	0.54	0.60	171.4205303	171.42	23.81	24.92	0.68	0.51	

Figure S30. Experimental and GIAO NMR calculated data for 3*R**,4*S**,7*S**-**1** (isomer 1) and 3*R**,4*S**,7*R**-**1** (isomer 2) following the STS method. (a) Data were generated following the STS method without further dichloro correction. (b) Data were generated following the STS method with further correction for dichloro carbon (marked in red).

(a)

Note:
Pmean > 5% is the confidence interval

Note:
sp carbons include C≡N
sp2-CH/C for double bonds except carbonyl group
C=O includes only ketones and aldehydes

	Isomer 1	Isomer 2
MAE	1.35	1.35
RMS	1.63	1.61
P_{mean}	19.12%	19.84%
Prel	40.80%	59.20%

				Isomer 1						Isomer 2						
Heavy atom beari	Type of carbon	Carbon no.	Exptl δ	Shielding tens	Corr Shielding	Calcd δ	Calcd δ (S(	abs dev	P	Shielding tens	Corr Shielding	Calcd δ	Calcd δ (S(	abs dev	P	Sl
C-Cl	sp3-CH2/CH/C		78.4	106.4037625	115.7754745	80.95	81.43	3.03	0.01	106.5917962	115.95	80.78	81.20	2.80	0.01	
C-Cl	sp3-CH2/CH/C		71.8	116.2302597	124.6409403	71.85	72.33	0.53	0.63	116.0448467	124.47	72.03	72.46	0.66	0.55	
	sp2-CH		134.5	66.10566204	66.10566204	132.71	133.14	1.36	0.38	65.93316549	65.93	132.87	133.24	1.26	0.41	
	sp2-CH		129.8	68.24832935	68.24832935	130.71	131.15	1.35	0.38	68.11119355	68.11	130.84	131.21	1.41	0.36	
C-Cl	sp3-CH2/CH/C		67.9	123.9684263	131.6223142	64.69	65.17	2.73	0.01	123.9459762	131.60	64.71	65.15	2.75	0.01	
C-Cl	sp3-CH2/CH/C		71.7	114.9072949	123.4473614	73.08	73.56	1.86	0.09	114.8281229	123.38	73.15	73.58	1.88	0.09	
	sp2-CH		139.5	60.74881305	60.74881305	137.70	138.13	1.37	0.37	60.80547012	60.81	137.65	138.01	1.49	0.34	
	sp2-CH2		116.5	80.70268373	80.70268373	115.85	116.29	0.21	0.63	80.60929049	80.61	115.94	116.32	0.18	0.68	
	sp3-CH3		25	171.4031183	171.4031183	23.83	24.35	0.65	0.53	171.1996226	171.20	24.04	24.52	0.48	0.64	
	sp3-CH3		24.9	171.307225	171.307225	23.93	24.45	0.45	0.66	171.4205303	171.42	23.81	24.29	0.61	0.56	

(b)

Note:
Pmean > 5% is the confidence interval

Note:
sp carbons include C≡N
sp2-CH/C for double bonds except carbonyl group
C=O includes only ketones and aldehydes

	Isomer 1	Isomer 2
MAE	1.18	1.23
RMS	1.43	1.49
P_{mean}	25.80%	23.57%
Prel	71.24%	28.76%

				Isomer 1						Isomer 2						
Heavy atom beari	Type of carbon	Carbon no.	Exptl δ	Shielding tens	Corr Shielding	Calcd δ	Calcd δ (S(	abs dev	P	Shielding tens	Corr Shielding	Calcd δ	Calcd δ (S(	abs dev	P	S
None	sp3-CH2/CH/C		78.4	121.1445606	121.1445606	75.44	76.49	1.91	0.09	121.306514	121.31	75.28	76.28	2.12	0.06	
C-Cl	sp3-CH2/CH/C		71.8	116.2302597	124.6409403	71.85	72.91	1.11	0.32	116.0448467	124.47	72.03	73.04	1.24	0.26	
	sp2-CH		134.5	66.10566204	66.10566204	132.71	133.59	0.91	0.56	65.93316549	65.93	132.87	133.68	0.82	0.59	
	sp2-CH		129.8	68.24832935	68.24832935	130.71	131.60	1.80	0.24	68.11119355	68.11	130.84	131.66	1.86	0.23	
C-Cl	sp3-CH2/CH/C		67.9	123.9684263	131.6223142	64.69	65.77	2.13	0.05	123.9459762	131.60	64.71	65.74	2.16	0.05	
C-Cl	sp3-CH2/CH/C		71.7	114.9072949	123.4473614	73.08	74.13	2.43	0.03	114.8281229	123.38	73.15	74.16	2.46	0.03	
	sp2-CH		139.5	60.74881305	60.74881305	137.70	138.57	0.93	0.54	60.80547012	60.81	137.65	138.44	1.06	0.49	
	sp2-CH2		116.5	80.70268373	80.70268373	115.85	116.78	0.28	0.52	80.60929049	80.61	115.94	116.80	0.30	0.49	
	sp3-CH3		25	171.4031183	171.4031183	23.83	25.03	0.03	0.98	171.1996226	171.20	24.04	25.22	0.22	0.83	
	sp3-CH3		24.9	171.307225	171.307225	23.93	25.13	0.23	0.82	171.4205303	171.42	23.81	24.99	0.09	0.93	

Figure S31. Experimental and GIAO NMR calculated data for 3*R**,4*S**,7*S**-**2** (isomer 1) and 3*R**,4*S**,7*R**-**2** (isomer 2) following the STS method. (a) Data were generated following the STS method without further dichloro correction. (b) Data were generated following the STS method with further correction for dichloro carbon (marked in red).

(a)

Note:
Pmean > 5% is the confidence interval

Note:
sp carbons include C≡N
sp2-CH/C for double bonds except carbonyl group
C=O includes only ketones and aldehydes

Isomer 1	
MAE	4.69
RMS	7.14
P_{mean}	#NUM!
Prel	#NUM!

Isomer 2	
MAE	6.60
RMS	8.93
P_{mean}	#NUM!
Prel	#NUM!

Heavy atom beari	Type of carbon	Carbon no.	Exptl δ	Isomer 1: Shielding tens	Corr Shielding	Calcd δ	Calcd δ (SC	abs dev	P	Isomer 2: Shielding tens	Corr Shielding	Calcd δ	Calcd δ (SC	abs dev	P
None	sp2-CH2		116.5	80.41362258	80.41362258	116.13	114.27	2.23	0.00	80.58313663	80.58	115.96	113.66	2.84	0.00
None	sp2-CH		139.5	61.61190888	61.61190888	136.90	136.26	3.24	0.04	61.1942226	61.19	137.29	136.23	3.27	0.03
C-Cl	sp3-CH2/CH/C		71.9	115.0610618	123.58609	72.94	68.55	3.35	0.00	115.2529073	123.76	72.76	67.95	3.95	0.00
C-Cl	sp3-CH2/CH/C		68.8	120.4282047	128.4283263	67.97	63.28	5.52	0.00	120.1858022	128.21	68.19	63.11	5.69	0.00
None	sp2-CH		130.2	68.36281881	68.36281881	130.61	129.60	0.60	0.70	65.37133991	65.37	133.39	132.11	1.91	0.22
None	sp2-CH		128.3	70.03796712	70.03796712	129.05	127.95	0.35	0.82	75.0720184	75.07	124.36	122.55	5.75	0.00
None	sp2-C		138.5	60.16600267	60.16600267	139.08	138.58	0.08	0.97	61.99002443	61.99	137.29	136.24	2.26	0.24
C-Cl	sp2-CH		117.2	70.5105394	76.6679155	122.87	121.41	4.21	0.01	66.35225454	72.58	126.68	125.00	7.80	0.00
None	sp3-CH3		25.1	171.0685685	171.0685685	24.18	16.93	8.17	0.00	171.6924108	171.69	23.53	15.86	9.24	0.00
C-Br	sp3-CH2/CH/C		33.7	124.0980213	138.0137334	58.13	52.87	19.17	0.00	119.8368788	133.84	62.41	56.99	23.29	0.00

(b)

Note:
Pmean > 5% is the confidence interval

Note:
sp carbons include C≡N
sp2-CH/C for double bonds except carbonyl group
C=O includes only ketones and aldehydes

Isomer 1	
MAE	1.57
RMS	2.20
P_{mean}	11.24%
Prel	100.00%

Isomer 2	
MAE	3.17
RMS	3.90
P_{mean}	0.51%
Prel	0.00%

Heavy atom beari	Type of carbon	Carbon no.	Exptl δ	Isomer 1: Shielding tens	Corr Shielding	Calcd δ	Calcd δ (SC	abs dev	P	Isomer 2: Shielding tens	Corr Shielding	Calcd δ	Calcd δ (SC	abs dev	P
None	sp2-CH2		116.5	80.41362258	80.41362258	116.13	115.47	1.03	0.03	80.58313663	80.58	115.96	115.13	1.37	0.01
None	sp2-CH		139.5	61.61190888	61.61190888	136.90	136.28	3.22	0.04	61.1942226	61.19	137.29	136.57	2.93	0.06
C-Cl	sp3-CH2/CH/C		71.9	115.0610618	123.58609	72.94	72.19	0.29	0.80	115.2529073	123.76	72.76	71.67	0.23	0.83
C-Cl	sp3-CH2/CH/C		68.8	120.4282047	128.4283263	67.97	67.20	1.60	0.15	120.1858022	128.21	68.19	67.07	1.73	0.12
None	sp2-CH		130.2	68.36281881	68.36281881	130.61	129.98	0.22	0.89	65.37133991	65.37	133.39	132.66	2.46	0.11
None	sp2-CH		128.3	70.03796712	70.03796712	129.05	128.42	0.12	0.94	75.0720184	75.07	124.36	123.57	4.73	0.00
None	sp2-C		138.5	60.16600267	60.16600267	139.08	138.48	0.02	0.99	61.99002443	61.99	137.29	136.58	1.92	0.32
C-Cl	sp2-CH		117.2	70.5105394	76.6679155	122.87	122.23	5.03	0.00	66.35225454	72.58	126.68	125.90	8.70	0.00
None	sp3-CH3		25.1	171.0685685	171.0685685	24.18	23.32	1.78	0.09	171.6924108	171.69	23.53	22.15	2.95	0.00
None	sp3-CH2/CH/C		33.7	158.6274614	158.6274614	36.96	36.13	2.43	0.03	155.981718	155.98	39.68	38.40	4.70	0.00

Figure S32. Experimental and GIAO NMR calculated data for 7*Z*-**3** (isomer 1) and 7*E*-**3** (isomer 2) following the STS method. (a) Data were generated following the STS method without further dibromo correction. (b) Data were generated following the STS method with further correction for dibromo carbon (marked in red).

Table S1. ^{1}H and ^{13}C NMR spectroscopic data of **3**, **4**, and **5** in acetond-d_6.

	**3**[a]		**4**[b]		**5**[b]	
Position	δ_C (type)	δ_H (J in Hz)	δ_C (type)	δ_H (J in Hz)	δ_C (type)	δ_H (J in Hz)
1	117.0 (CH_2)	5.31 d (10.8)<br>5.47 d (17.2)	116.9 (CH_2)	5.30 d (11.0)<br>5.46 d (17.0)	117.1 (CH_2)	5.31 d (10.8)<br>5.48 d (16.8)
2	140.4 (CH)	6.15 dd (17.2, 10.8)	140.4 (CH)	6.16 dd (17.0, 11.0)	140.4 (CH)	6.06 dd (16.8, 10.8)
3	73.4 (C)		73.4 (C)		73.7 (C)	
4	70.0 (CH)	4.95 d (8.0)	70.0 (CH)	4.92 d (8.5)	70.0 (CH)	4.92 d (8.4)
5	131.1 (CH)	6.48 dd (15.2, 8.0)	131.2 (CH)	6.45 dd (16.0, 8.5)	131.3 (CH)	6.46 dd (16.0, 8.0)
6	129.1 (CH)	6.55 d (15.2)	127.7 (CH)	6.52 d (16.0)	127.7 (CH)	6.53 d (16.0)
7	139.3 (C)		138.3 (C)		138.4 (C)	
8	119.2 (CH)	6.64 s	121.4 (CH)	6.81 s	119.2 (CH)	6.64 s
9	26.1 (CH_3)	1.82 s	26.0 (CH_3)	1.81 s	27.4 (CH_3)	1.80 s
10	34.9 (CH)	7.19 s	66.9 (CH)	7.25 s	66.9 (CH)	7.26 s

[a] ^{13}C and ^{1}H spectroscopic data were recorded at 100 and 400 MHz, respectively. [b] ^{13}C and ^{1}H spectroscopic data were recorded at 125 and 500 MHz, respectively.

Table S2. Comparison of specific optical rotations and selected NMR data of synthetic halogenated monoterpenes.[a]

structure	$[\alpha]_D$ ($CHCl_3$)	δ_H (H-6)	δ_C (C-9)
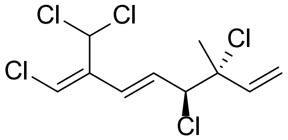	–6.2 (synthetic)	6.34–6.35 ($CDCl_3$)	25.2 ($CDCl_3$)
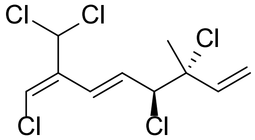	–32.0 (synthetic)	6.63 ($CDCl_3$)	25.2 ($CDCl_3$)
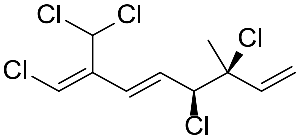	–28.3 (synthetic)	6.33–6.34 ($CDCl_3$)	27.7 ($CDCl_3$)
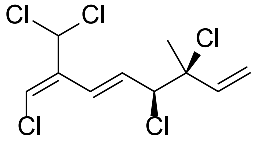	–58.8 (synthetic)	6.63 ($CDCl_3$)	27.7 ($CDCl_3$)
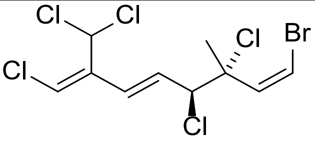	–32.1 (synthetic)	6.33 ($CDCl_3$)	26.4 ($CDCl_3$)
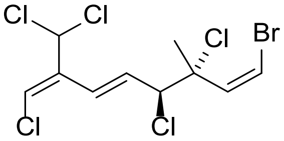	–28.7 (synthetic)	6.78 ($CDCl_3$)	26.4 ($CDCl_3$)

[a] Vogel et al. *Angew. Chem. Int. Ed.* **2014**, *53*, 12205–12209.
